# Supplementary material for: Redox Signaling by the RNA Polymerase III TFIIB-Related Factor Brf2
Source: Cell. 2015 Dec 3;163(6):1375–87. doi: 10.1016/j.cell.2015.11.005 (PMC4671959; doi:10.1016/j.cell.2015.11.005)
Supplement: Document S1. Supplemental Experimental Procedures and Tables S1 and S2 [file mmc1.pdf]

Cell

Supplemental Information

Redox Signaling by the RNA Polymerase III TFIIB-Related Factor Brf2

Jerome Gouge, Karishma Satia, Nicolas Guthertz, Marcella Widya, Andrew James Thompson, Pascal Cousin, Oleksandr Dergai, Nouria Hernandez, and Alessandro Vannini

SUPPLEMENTAL EXPERIMENTAL PROCEDURES

Expression and purification of recombinant proteins.

All proteins were expressed in Rosetta(DE3)pLysS. TB, and TBP-Brf2 complex were both expressed overnight at 20°C with 1 mM IPTG (Isopropyl β-D-1-thiogalactopyranoside) in TB medium. Full-length Brf2 was expressed at 30°C for 4 hours with 1 mM IPTG. Cells were grown at 37°C until the optical density reached 0.6 (A600), then the temperature was reduced and IPTG was then added 30 minutes after. Cells were harvested by centrifugation at 4000g (20 minutes at 4°C) and the pellets stored at -80°C. For crystallization, pellets of Brf2 co-expressed with TBP were re-suspended in 750 mM NaCl, 10 mM imidazole, 50 mM HEPES pH 7.9, 10% glycerol, 5 mM β-mercaptoethanol (buffer A1), supplemented with protease inhibitors tablets (Pierce) and DNase I (20 mg/ml). Cells were lysed through sonication and clarified by centrifugation (14000g for 45 minutes at 4°C). The supernatant was applied onto a 5 mL HisTrap HP column (GE healthcare) and bound proteins were washed with 50 ml of buffer A1 supplemented with 50 mM imidazole, then eluted with buffer B1 (buffer A1 containing 300 mM imidazole). The salt concentration was reduced to 350 mM by diluting the sample with buffer H1 (50 mM HEPES pH 7.9, 10% glycerol, 5 mM DTT). The proteins's samples were loaded onto a 5 mL HiTrap Heparin HP column (GE Healthcare) and eluted with a 375 mM to 1.5 M NaCl gradient in 10 column volumes. At this stage, Brf2-TBP complex was dissociating and the subsequent steps were performed to purify Brf2 in isolation. The fractions containing the protein of interest were pooled and the N-terminal His-tag was cleaved overnight at 4°C with the 3C protease. Imidazole (20 mM) was added to the sample before loading onto a 5 ml HisTrap HP column. The flow-through was concentrated and applied onto a Superdex 200 26/600 column (GE Healthcare). The size exclusion chromatography was performed in a buffer containing 500 mM ammonium acetate, 50 mM HEPES pH 7.9, 10% glycerol and 2 mM DTT (gel filtration buffer). The fractions containing Brf2 were pooled, concentrated, flash-frozen and stored at -80°C. For full-length Brf2, the same protocol was used, with the exception that the tag was not cleaved and the second nickel column step was omitted. For experiments aiming to assess the effect of oxidative modifications on Brf2, DTT was removed from the buffer though a PD-10 desalting column (GE Healthcare) and the protein was immediately flash frozen.

His-TBP pellets were re-suspended in buffer A2 (A1 buffer containing 500 mM NaCl) supplemented with protease inhibitors tablets (Pierce) and DNase I (20 mg/ml). Cell lysis and clarification were performed as mentioned above and the supernatant applied on a 5 ml HisTrap HP column (GE healthcare). The bound proteins were washed with 50 ml of buffer A2 supplemented with 50 mM imidazole, then eluted with buffer B2 (similar to buffer B1 but with 500 mM NaCl). The fractions containing His-TBP were pooled and loaded onto a 5mL HiTrap Heparin HP column (GE Healthcare). The protein was eluted with 500 mM to 2 M NaCl gradient in 10 column volumes. The tag was cleaved overnight at 4°C by addition of the 3C protease. The reaction was then adjusted to 20 mM

imidazole and loaded onto a HisTrap HP column. The flow through was concentrated and loaded onto a Superdex 200 16/600 column (GE Healthcare) in gel filtration buffer. After elution, the protein was concentrated, flash frozen and stored at -80°C. SNAPc was expressed in insect cells and purified as described (Henry et al., 1998).

Oligonucleotides.

All oligonucleotides were purchased from Integrated DNA Technologies. Each single strand was re-suspended in 50 mM Tris pH 8.0, 5 mM $MgCl_2$, 1 mM EDTA and mixed with the complementary strand in an equimolar ratio, heated at 95°C for 3 minutes. then allowed to cool down to room temperature overnight.

Crystallization, data collection and processing.

DNA sequences used for crystallization are described in Figure S1. The complexes were assembled at a final concentration of 60 μM with an equimolar ratio of TBP and Brf2 and 1.2 excess of the double stranded DNA. Crystals were grown by mixing 1 μL of the complexes and 1 μL of the crystallization solution (10-20% PEG 3350, 50-100 mM $MgCl_2$, 2 mM DTT) in hanging drop plates. Seeds were introduced after 3 hours of equilibration. After 5 days, crystals were cryo-protected in a mix of 50% paraffin/50% paratone and flash frozen in liquid nitrogen. Initial data sets were collected at the ESRF (France) on ID29 and ID23eh1. The final high-resolution diffraction data were collected on beamlines I24 (U6) and IO3 (RPPH1 and TRNAU1) at Diamond Light Source (UK). The data were indexed with XDS (Kabsch, 2010), scaled and merged with SCALA (Evans, 2011) from the ccp4 package (Winn et al., 2011). The data were processed using $CC_{1/2}$ and completeness as cut-off criterion (Karplus and Diederichs, 2012).

Structure determination and refinement.

The structure of Brf2 bound to TBP and a U6 promoter was solved by molecular replacement using the TFIIB structure (PDB id: 1C9B) as a search model in PHASER (McCoy et al., 2007). The solution was confirmed by using a 2 ensemble search approach (first TBP and dsDNA then the cyclin repeats of TFIIB). The quality of the resulting electronic density did not allow the manual reconstruction directly after the molecular replacement. To improve the phases, solvent flattening and dummy atom building were carried out with DM (Cowtan, 2010) and ARP/wARP (Langer et al., 2008), respectively. The density modified maps clearly showed α-helices in the cyclin repeats region. Iterative manual building was performed with COOT (Emsley et al., 2010), and BUSTER-TNT (version 2.10.1) was used for refinement (Blanc et al., 2004). The U6-2 structure was used a search model for molecular replacement of RPPH1 and TRNAU1 structures. The quality of the final structures was assessed with MolProbity (Chen et al., 2010). Data collection and refinement statistics are shown in Table S2. The figures were prepared with PyMol (version 1.7.0.5 Schrödinger, LLC). The alignments were

performed with Clustal Omega (Sievers and Higgins, 2014) and prepared with ESpript (Robert and Gouet, 2014).

Electrophoretic mobility shift assay.

The oligonucleotides used in this study are all Cy-5 labelled and based on the U6_2 sequence: 5'-Cy5- ATTTGATTGAAGGGCTTAAAATAGGTGTGACAGTAACC-3' and 5'-GGTTACTGTCACACCTATTTTAAGCCCTTCAATCAAAT-3'. The complexes were assembled in a 25 µL reaction volume in a buffer containing 500 mM ammonium acetate, 50 mM HEPES pH 7.9, 10% glycerol with or without the addition of 5 mM DTT. The complexes were assembled by mixing 1 pmol of the dsDNA with 75 pmol of TBP and 25 pmol of Brf2 and incubated at room temperature for 1 hour. For the EMSAs in Figure 5, 10 pmol of Brf2, from which the DTT had been removed with a PD-10 column, was pre-incubated with either H_2O_2, or oxidised/reduced glutathione (GSSG/GSH) gradients (3 m final) for 20 minutes at room temperature. Then 15 pmol of TBP and 1 pmol of dsDNA (with 1.1 excess of labeled ssDNA) were added to a final volume of 25 µL. The incubation lasted for 20 minutes. For the incubation with iodoacetamide, Brf2 (10 pmol) was first reduced with 0.5 mM DTT for 15 minutes, then the iodoacetamide was added at the final concentration indicated. The reaction was quenched after 30 minutes at room temperature with 50 mM DTT. TBP and the DNA were then added as previously described. For the time course experiment, 10 pmol of Brf2 were incubated at 4°C for different time points, then TBP and the DNA were added. The binding reactions were resolved on a 4% polyacrylamide (37.5:1 acrylamide/bis-acrylamide, 10% glycerol, Tris borate EDTA 1X) gel in 1X Tris- borate EDTA running buffer at 40 mA. The gels were then scanned with a Typhoon FLA 9500 and band quantification was carried out with ImageQuanT TL version 8.1. The EMSA with SNAPc (Figure S3A) was performed as described previously (Saxena et al., 2005).

Pull-downs.

To study the interaction of Brf2 and TBP, 50 µg of Brf2 were loaded onto His SpinTrap columns (GE Healthcare) in presence or in absence of 100 µg of TBP, in a buffer containing 500 mM ammonium acetate, 50 mM HEPES pH 7.9, 10 mM imidazole, 10% glycerol and 2 mM β-mercaptoethanol in a total volume of 400 µL. The incubation was performed at 4°C for 2 hours. The beads were extensively washed with the same buffer supplemented with 50 mM imidazole. The proteins were eluted with 75 µL of the binding buffer containing 300 mM imidazole, boiled and analyzed on a SDS-PAGE.

Fluorescence polarization assay.

The oligonucleotides used for the fluorescence polarization studies, 5'-Alexa488-ATTGAAGGGCTTAAAATAGGTGTGACAG-3' and 5'-GGTTACTGTCACACCTATTTTAAGCCCTTCAATCAAAT-3', were purchased from Integrated DNA Technologies, annealed overnight as previously described. The TBP-DNA complex was assembled

by saturating the oligonucleotides (100 nM final) with 250 mM final of TBP at room temperature for an hour in a volume of 5 µL. A serial dilution of Brf2 (from 10 mM to 5nM) was added and incubated for an extra hour at room temperature in 50 µL total reaction volume. The data were collected at 25°C on a POLARstar Omega plate reader (BMG Labtech), with excitation at 485 nm and emission at 520 nm, and analyzed with MARS Data Analysis Software version 2.10 R3 (BMG Labtech). The binding constants were calculated with GraphPad Prism 6. Three independent experiments were carried out in order to calculate standard deviation.

Mass spectrometry.

Solvents were purchased from Rathburn (Walkerburn, UK). All other reagents were purchased from Sigma unless otherwise stated. All the mass spectrometry experiments were performed with Brf2 full-length carrying a His-Tag at its C-terminus. Prior to treatment, the buffer was exchanged with PD-10 columns (GE Healthcare) to have Brf2 in 50 mM HEPES pH 7.9 and 500 mM ammonium acetate. To check the different oxidative intermediates, Brf2 was incubated at room temperature for 1 hour with a) 5 mM DTT, b) 3 mM of oxidized glutathione or c) overnight with 2 mM dimedone. Solutions of 10-20 µM Brf2 were diluted 3-4 fold with 50 mM triethylammonium bicarbonate/5% acetonitrile and digested directly by addition of 20-50 ng trypsin and incubation for 1 hour at 37°C, followed by a second addition of 20-50 ng trypsin and incubation for a further 3 hours at 37°C. A 5 µL aliquot of each digest was injected directly for LC-MS analysis. Reversed phase chromatography was performed with an HP1200 platform (Agilent). Peptides were resolved on a 75 µm I.D. 15 cm C18 packed emitter column (3 µm particle size; Nikkyo Technos) over 30 minutes using a linear gradient of 96:4 to 50:50 buffer A:B (buffer A: 1% acetonitrile/3% dimethyl sulfoxide/0.1% formic acid; buffer B: 80% acetonitrile/3% dimethyl sulfoxide/0.1% formic acid) at 250 nL/minute. Peptides were ionised by electrospray ionisation with 1.8 kV applied immediately pre-column via a microtee built into the nanospray source. Sample was infused into an LTQ Velos Orbitrap mass spectrometer (Thermo Fisher Scientific) directly from the end of the tapered tip silica column (6-8 µm exit bore). The ion transfer tube was heated to 275°C and the S-lens set to 60%. MS/MS were acquired using data dependent acquisition based on a full 30,000 resolution FT-MS scan with preview mode disabled and internal lock mass calibration against ion 401.922718. The top 10 most intense ions were fragmented by collision-induced dissociation and analyzed with enhanced ion trap scans. Precursor ions with unknown or single charge states were excluded from selection. Automatic gain control was set to 1,000,000 for FT-MS and 30,000 for IT-MS/MS, full FT-MS maximum inject time was 500 ms and normalized collision energy was set to 35% with an activation time of 10 ms. Wideband activation was used to co-fragment precursor ions undergoing neutral loss of up to -20 m/z from the parent ion, including loss of water/ammonia. MS/MS was acquired for selected precursor ions with a single repeat count acquired after 5 seconds delay followed by dynamic exclusion with a 10 ppm mass window for 10 seconds based on a maximal exclusion list of 500 entries. Raw MS/MS data was compiled and interrogated against the swissprot 2011_01 human database, customized to include the

Brf2 construct sequence, with Proteome Discoverer v1.4 and Mascot v2.3. The Mascot search parameters used were: 10 ppm precursor peptide mass error, 0.25 Da fragment mass error, and trypsin specificity with up to two missed cleavages.

Cell culture.

MRC5 and A549 cell lines (ATCC, UK) were maintained and grown in EMEM (ATCC, UK) and Hams F-12 (Kaighn's) media (Life technologies, CA, USA) respectively, and supplemented with 10% (v/v) FBS (Life technologies) and 100 U/ml penicillin and streptomycin (Sigma). MCF10A (ATCC, UK) were maintained in DMEM/F12 (Life technologies, CA, USA) and supplemented with 5% (v/v) donor horse serum (16050122, Life technologies), Insulin (10 µg/ml, I9278, sigma), cholera toxin (100 ng/ml, C8052, Sigma), EGF (20 ng/ml, AF-100-15, Peprotech), Hydrocortisone (0.5 mg/ml, H6909, Sigma) and 100 U/ml penicillin and streptomycin. All cells were grown at 37°C in a 5% CO2 humidified atmosphere. Cells were treated with varying concentrations of t-BHP (Sigma, St Louis, MA, USA) in the conditioned medium and subsequently lysed with RIPA lysis buffer (50 mM Tris pH 8.0, 150 mM NaCl, 1% (v/v) NP40, 0.5% (v/v) Sodium deoxycholate, 2 mM EDTA) containing protease inhibitors (Pierce).

Immunoprecipitation.

Brf2 antibodies (ab17011, Abcam) were coupled to magnetic beads using the DynaBeads Antibody coupling kit (Life Technologies) according to the manufacturer's protocol. After lysis and centrifugation, the protein concentration in the supernatant was measured with Bradford Protein Assay (Bio-Rad) to ensure that the same amount of total proteins was incubated with the beads. The cell lysates were then incubated overnight at 4°C. Immunoprecipitates were extensively washed with lysis buffer and eluted with SDS loading buffer by heating at 50°C for 10 minutes.

Quantitative RT-PCR.

Total RNA was extracted from treated cells with TRIzol reagent (Life technologies) according to manufacturer's instructions. The Quantitect reverse transcription kit (Qiagen, Netherlands) was used to synthesize cDNA following manufacturers instructions, except for a 10 minutes incubation step at 70°C before addition of the polymerase to the reaction. To determine levels of selenocysteine precursors and mature tRNA, RPPH1 (PPH68975A-200, Qiagen), RNA7SK (PPH68975A-200, Qiagen), U6, tRNA Leu and 5S rRNA, syber green assays were performed. Primers 1) 5'-TCAGTGGTCTGGGGTGCAGG-3' and, 2) 5'- GTCCGGTTCGATAAGTAAGATTTAAGGC-3' were used to determine selenocysteine tRNA precursor levels. Primers 1) 5'-GCACCCCAGACCACTGAG GA-3' and Primers 2) 5'-AGCGACAGAGTGGTTCAATTCCAC-3' were used to determine mature selenocysteine tRNA levels. Primers 1) 5'-GGCCATACCACCCTGAACGC-3' and 2) 5'-CAGCACCCGGTATTCCCAGG-3 were used to determine 5S rRNA levels, the reference transcript for all syber green assays (loading control). Primers used to determine levels of U6 were 1) 5'-

GGAACTCGAGTTTGCGTGTCATCCTTGCGC-3 2) 5’-GGAATCTAGAACATATACTAAAATTGGAAC-3’ and tRNA Leu were 1) 5’-GTCAGGATGGCCGAGTGGTCTAA-3’ 2) 5’-TGTCAGAAGTGGGATTCGAACCCAC-3’. Amplification of cDNA was performed in a 20 µl reaction containing either 1) PerfeCTa SYBR Green FastMix (VWR, Radnor, USA), 0.3 µM of syber green primers mentioned above, and cDNA, (22.5 and 15 ng to detect selenocysteine tRNA precursor and mature levels resepctively and 7.5 ng of cDNA for other RNA levels). RT-PCR was performed with the Applied Biosystems 7900HT Fast Real time PCR system and the samples were subjected to an initial denaturation step at 95°C for 10 minutes, followed by 40-cycles of 95°C for 20s, 60°C for 20s, and 72°C for 20s. All RNA levels were quantified with the relative standard curve method and the Brf1-dependent 5S rRNA as an internal control. Mature selenocysteine tRNA levels were quantified using the comparative Ct method and 5S rRNA as internal control. Prism 6 was used to perform unpaired t-test statistical analysis to determine p values. P values < 0.05 were deemed significant and data shown is from three biological repeats.

Flow cytometry.

Cells were trypsinized and re-suspended in media containing 1 µg mL-1 of Propidium iodide (Sigma), 5 µL per well of FITC-Annexin V (BD Biosciences) and 2.5 mM $CaCl_2$. Cells were then analyzed by flow cytometry on a LSR II SPOC instrument (BD Biosciences).

Western Blot.

Proteins were separated via SDS-PAGE and transferred to a nitrocellulose membrane (GE-Healthcare). The membrane was then blocked for 1 hour in protein-free RotiBlock buffer (Carl Roth, Germany) and then incubated for 1 hour at room temperature with primary antibodies for anti-BRF2 (ab17011, 1:1000, Abcam), anti-beta Actin (ab6276, 1:5000, Abcam) or anti-Glutathione (ab19534, 1:1000, Abcam) diluted in RotiBlock buffer. In the case of anti-GPX1 (ab50427, 1:500, Abcam), anti-GPX2 (ab137431, 1:1000, Abcam), anti-GPX4 (ab125066, 1:1000, Abcam), anti-SEP15 (ab124840, 1:1000, Abcam), anti-SelM (ab133681, 1:1000 Abcam), anti-Nrf2 (ab62352, 1:1000, Abcam) primary incubations were performed overnight at 4°C in 5% (w/v) BSA TBS-T. The membranes were then washed with TBST three times, followed by incubation with either rabbit anti Goat IgG-HRP (sc-2768, 1:5000, Santa Cruz Biotechnology, Santa Cruz USA), ECL peroxidase labelled anti-mouse (NA931, 1:5000, VWR) or anti-rabbit (NA931, 1:5000, VWR), and then developed.

Table S1, Related to Figure 1: Actively transcribed Brf2-dependent genes.

Name of gene	Description	Genome location	Function
RMRP	RNA component of mitochondrial RNA processing endoribonuclease	NC_000009.12 /9p21-p12	Mitochondrial RNA processing
RN7SK	RNA polymerase II regulation	NC_000006.12 /6p12.2	P-TEFb (RNA Pol II elongation) inhibition
RNU6 (U6-1)	Component of the spliceosome, catalytic RNA	NC_000015.10 /15q23	RNA processing
RNU6 (U6-2)	Component of the spliceosome, catalytic RNA	19p13.3	RNA processing
RNU6 (U6-7)	Component of the spliceosome, catalytic RNA	NC_000014.9 /14q12	RNA processing
RNU6 (U6-8)	Component of the spliceosome, catalytic RNA	NC_000014.9 /14q12	RNA processing
RNU6 (U6-9)	Component of the spliceosome, catalytic RNA	NC_000019.10 /19p13.3	RNA processing
RNU6ATAC	Component of the minor spliceosome	NC_000009.12 /9q34.2	RNA processing
RNY1	Component of the ribonucleoproteins Ro, implications in DNA replication	NC_000007.14 /7q36	DNA replication
RNY3	Component of the ribonucleoproteins Ro, implications in DNA replication	NC_000007.14 /7q36	DNA replication
RNY4	Component of the ribonucleoproteins Ro, implications in DNA replication	NC_000007.14 /7q36	DNA replication
RNY5	Component of the ribonucleoproteins Ro, implicated in DNA replication	NC_000007.14 /7q36	DNA replication
RPPH1	Component of the RNase P, implicated in tRNA maturation	NC_000014.9/ 14q11.2	5’-tRNA processing
TRNAU1	Selenocysteine tRNA	NC_000019.10/ 19q13.2-q13.3	Translation of selenoproteins

Table S2, Related to Figure 1: Data collection and refinement statistics

	U6 #2	RPPH1	TRNAU
**Data collection**			
Space group	$P2_12_12_1$	$P2_12_12_1$	$P2_12_12_1$
Cell dimensions			
a, *b*, *c* (Å)	77.3 89.9 102.5	77.7 91.9 102.4	77.0 91.3 102.9
a, *b*, *g* (°)	90.0 90.0 90.0	90.0 90.0 90.0	90.0 90.0 90.0
Resolution (Å)	29.5-1.9 (2.0-1.9) *	30-2.2 (2.3-2.2) *	30-2.7 (2.85-2.7) *
R_{merge}	0.058 (3.19) *	0.069 (1.617) *	0.157 (2.216) *
I/s*I*	11.2 (0.5) *	13.9 (0.8) *	6.9 (0.9) *
Completeness (%)	99.3 (99.3) *	99.7 (98.7) *	98.9 (99.4) *
Redundancy	4.2 (4.2) *	5.3 (4.3) *	4.8 (5.0) *
CC1/2	0.999 (0.287) *	0.999 (0.367) *	0.992 (0.312) *
**Refinement**			
Resolution (Å)	29.5-1.9	29.7-2.2	29.7-2.7
No. reflections	56427	199497	20221
$R_{work/}$ R_{free} (%)	18.6/21.6	23.6/25.8	21.4/24.9
No. atoms			
Protein	3832	3826	3798
DNA	1101	1088	1122
Water	388	201	141
B-factors			
Protein	56.9	68.0	79.8
DNA	79.9	86.3	90.5
Water	58.8	59.4	60.5
R.m.s deviations			
Bond lengths (Å)	0.010	0.009	0.010
Bond angles (º)	0.94	0.96	0.99

*Highest resolution shell is shown in parenthesis.

SUPPLEMENTAL REFERENCES

Bailey, T.L., Boden, M., Buske, F.A., Frith, M., Grant, C.E., Clementi, L., Ren, J., Li, W.W., and Noble, W.S. (2009). MEME SUITE: tools for motif discovery and searching. Nucleic Acids Res *37*, W202-208.

Blanc, E., Roversi, P., Vonrhein, C., Flensburg, C., Lea, S.M., and Bricogne, G. (2004). Refinement of severely incomplete structures with maximum likelihood in BUSTER-TNT. Acta crystallographica Section D, Biological crystallography *60*, 2210-2221.

Chen, V.B., Arendall, W.B., 3rd, Headd, J.J., Keedy, D.A., Immormino, R.M., Kapral, G.J., Murray, L.W., Richardson, J.S., and Richardson, D.C. (2010). MolProbity: all-atom structure validation for macromolecular crystallography. Acta crystallographica Section D, Biological crystallography *66*, 12-21.

Cowtan, K. (2010). Recent developments in classical density modification. Acta crystallographica Section D, Biological crystallography *66*, 470-478.

Emsley, P., Lohkamp, B., Scott, W.G., and Cowtan, K. (2010). Features and development of Coot. Acta crystallographica Section D, Biological crystallography *66*, 486-501.

Evans, P.R. (2011). An introduction to data reduction: space-group determination, scaling and intensity statistics. Acta crystallographica Section D, Biological crystallography *67*, 282-292.

Henry, R.W., Mittal, V., Ma, B., Kobayashi, R., and Hernandez, N. (1998). SNAP19 mediates the assembly of a functional core promoter complex (SNAPc) shared by RNA polymerases II and III. Genes and Development *12*, 2664-2672.

Honda, S., Shigematsu, M., Morichika, K., Telonis, A.G., and Kirino, Y. (2015). Four-leaf clover qRT-PCR: A convenient method for selective quantification of mature tRNA. RNA Biol *12*, 501-508.

Kabsch, W. (2010). Xds. Acta crystallographica Section D, Biological crystallography *66*, 125-132.

Karplus, P.A., and Diederichs, K. (2012). Linking crystallographic model and data quality. Science *336*, 1030-1033.

Langer, G., Cohen, S.X., Lamzin, V.S., and Perrakis, A. (2008). Automated macromolecular model building for X-ray crystallography using ARP/wARP version 7. Nat Protoc *3*, 1171-1179.

McCoy, A.J., Grosse-Kunstleve, R.W., Adams, P.D., Winn, M.D., Storoni, L.C., and Read, R.J. (2007). Phaser crystallographic software. Journal of applied crystallography *40*, 658-674.

Robert, X., and Gouet, P. (2014). Deciphering key features in protein structures with the new ENDscript server. Nucleic Acids Res *42*, W320-324.

Saxena, A., Ma, B., Schramm, L., and Hernandez, N. (2005). Structure-function analysis of the human TFIIB-related factor II protein reveals an essential role for the C-terminal domain in RNA polymerase III transcription. Molecular and cellular biology *25*, 9406-9418.

Sievers, F., and Higgins, D.G. (2014). Clustal Omega, accurate alignment of very large numbers of sequences. Methods Mol Biol *1079*, 105-116.

Winn, M.D., Ballard, C.C., Cowtan, K.D., Dodson, E.J., Emsley, P., Evans, P.R., Keegan, R.M., Krissinel, E.B., Leslie, A.G., McCoy, A.*, et al.* (2011). Overview of the CCP4 suite and current developments. Acta crystallographica Section D, Biological crystallography *67*, 235-242.
