# Supplementary material for: Redox Signaling by the RNA Polymerase III TFIIB-Related Factor Brf2
Source: Cell. 2015 Dec 3;163(6):1375–87. doi: 10.1016/j.cell.2015.11.005 (PMC4671959; doi:10.1016/j.cell.2015.11.005)
Supplement: Document S2. Article plus Supplemental Information [file mmc2.pdf]

Cell

Article

Redox Signaling by the RNA Polymerase III TFIIB-Related Factor Brf2

Graphical Abstract

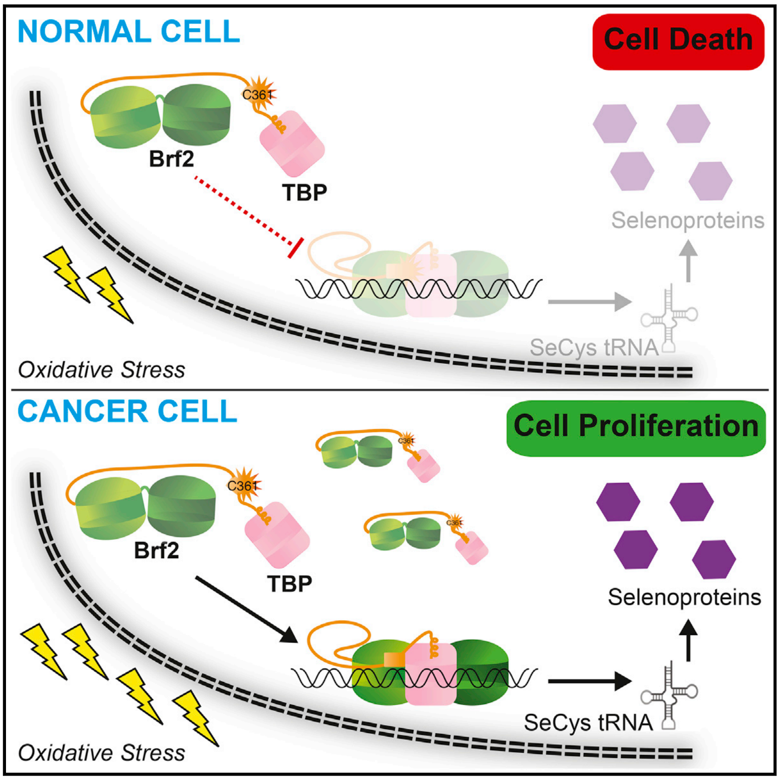

Authors

Jerome Gouge, Karishma Satia, Nicolas Guthertz, ..., Oleksandr Dergai, Nouria Hernandez, Alessandro Vannini

Correspondence

alessandro.vannini@icr.ac.uk

In Brief

Direct redox-sensing by the RNA polymerase III core transcription factor Brf2 couples cellular responses to oxidative stress and regulation of transcriptional output, contributing to the ability of cancer cells to evade death induced by reactive oxygen species.

Highlights

- Architectural conservation of TFIIB and TFIIB-related factors
- Brf2 is a redox-sensing RNA polymerase III core transcription factor
- Brf2 regulates cellular responses to oxidative stress
- Brf2 amplification enables cancer cells to evade oxidative stress-induced apoptosis

Accession Numbers

4ROC
4ROD
4ROE

Gouge et al., 2015, Cell *163*, 1375–1387
December 3, 2015 ©2015 The Authors
http://dx.doi.org/10.1016/j.cell.2015.11.005

Cell

Redox Signaling by the RNA Polymerase III TFIIB-Related Factor Brf2

Jerome Gouge,[1,3] Karishma Satia,[1,3] Nicolas Guthertz,[1] Marcella Widya,[1] Andrew James Thompson,[1] Pascal Cousin,[2] Oleksandr Dergai,[2] Nouria Hernandez,[2] and Alessandro Vannini[1,*]
[1]Division of Structural Biology, The Institute of Cancer Research, London SW7 3RP, UK
[2]Center for Integrative Genomics, Faculty of Biology and Medicine, University of Lausanne, 1015 Lausanne, Switzerland
[3]Co-first author
*Correspondence: alessandro.vannini@icr.ac.uk
http://dx.doi.org/10.1016/j.cell.2015.11.005
This is an open access article under the CC BY license (http://creativecommons.org/licenses/by/4.0/).

SUMMARY

TFIIB-related factor 2 (Brf2) is a member of the family of TFIIB-like core transcription factors. Brf2 recruits RNA polymerase (Pol) III to type III gene-external promoters, including the U6 spliceosomal RNA and selenocysteine tRNA genes. Found only in vertebrates, Brf2 has been linked to tumorigenesis but the underlying mechanisms remain elusive. We have solved crystal structures of a human Brf2-TBP complex bound to natural promoters, obtaining a detailed view of the molecular interactions occurring at Brf2-dependent Pol III promoters and highlighting the general structural and functional conservation of human Pol II and Pol III pre-initiation complexes. Surprisingly, our structural and functional studies unravel a Brf2 redox-sensing module capable of specifically regulating Pol III transcriptional output in living cells. Furthermore, we establish Brf2 as a central redox-sensing transcription factor involved in the oxidative stress pathway and provide a mechanistic model for Brf2 genetic activation in lung and breast cancer.

INTRODUCTION

In the eukaryotic nucleus, RNA polymerase (Pol) III transcribes genes encoding essential RNAs, including tRNAs and the 5S rRNA. The accurate recruitment of Pol III to its target genes and the formation of a transcriptionally active pre-initiation complex (PIC) occur through the association of Pol III with several specific transcription factors but TFIIIB is the key factor required for this process (Kassavetis et al., 1999; Schramm and Hernandez, 2002). TFIIIB is a complex composed of the TFIIB-related factor 1 (Brf1) (López-De-León et al., 1992; Wang and Roeder, 1995), the TATA binding protein (TBP) (Kassavetis et al., 1992; Lobo et al., 1992), and Bdp1, a SANT domain-containing protein (Kassavetis et al., 1995; Schramm et al., 2000). Vertebrates contain an alternative TFIIIB complex in which Brf1 is replaced by the TFIIB-related factor 2 (Brf2) (Cabart and Murphy, 2001; Schramm et al., 2000; Teichmann et al., 2000).

The Brf2-containing TFIIIB complex recruits Pol III to type III promoters, characterized by a TATA box located 20–25 base pairs (bp) upstream of the transcriptional start site and a proximal sequence element (PSE) located further upstream (Schramm and Hernandez, 2002). The TATA box is recognized by the Brf2-TBP complex, which binds synergistically with $SNAP_c$, a complex binding to the PSE (Henry et al., 1995). Actively transcribed Brf2-dependent genes have been characterized genome-wide (Carrière et al., 2012; James Faresse et al., 2012; Oler et al., 2010) and encode a small collection of RNAs including the spliceosomal U6 small nuclear RNA (snRNA), the RNA component of the tRNA processing enzyme RNase P, and the selenocysteine tRNA (Table S1).

Brf2 and Brf1 are part of a family of TFIIB-like transcription factors that share structural and functional features with the archetypal Pol II transcription factor TFIIB (Knutson and Hahn, 2011; Naidu et al., 2011; Vannini and Cramer, 2012). These factors all contain an N-terminal B-ribbon/reader/linker domain, which protrudes toward the polymerase active site, and a B-core domain consisting of two cyclin fold imperfect repeats, which in TFIIB binds simultaneously to the core of the Pol II enzyme, TBP, and the DNA. Additionally, Brf2 and Brf1 contain C-terminal domains (CTDs), which represent the major site of interaction with the adjoining TFIIIB subunits Bdp1 and TBP (Saxena et al., 2005). Whether TFIIB-like factors display the same architecture as TFIIB when bound to TBP and DNA is currently not known.

Pol III transcription is tightly regulated during the cell-cycle and its upregulation has been linked to tumorigenesis (White, 2011). Recently, Brf2 was found to be specifically amplified in the squamous cell carcinoma subtype of non-small cell lung cancer (Lockwood et al., 2010). Additionally, Brf2 overexpression is observed in several forms of cancers and correlates with poor patient survival rates, implicating Brf2 as a general oncogene, a prognosis marker, and a target for new anticancer therapies (Cabarcas and Schramm, 2011; Lu et al., 2013, 2014). Despite its significance, very little is known about the molecular architecture and mechanisms underlying Brf2-dependent Pol III transcription and its regulation.

RESULTS

Architecture of Brf2-TBP/DNA Complexes

We obtained X-ray crystallographic structures of Brf2-TBP complexes bound to the U6 snRNA (copy number 2, U6-2), RPPH1,

CrossMark

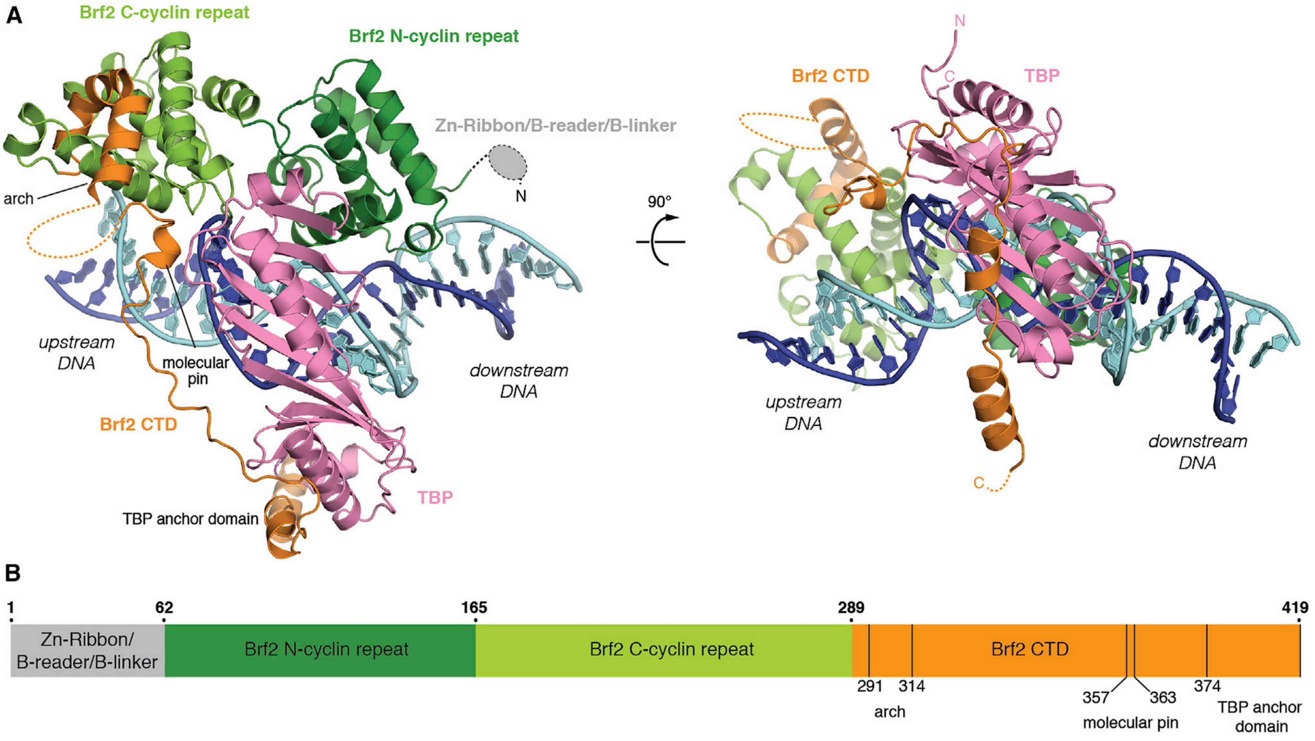

Figure 1. Structure of the Brf2-TBP/DNA Ternary Complex

(A) Overview of the Brf2-TBP/U6 promoter structure. DNA template and non-template strands are in blue and cyan respectively. Dashed lines represent disordered regions or regions not present in the crystallization construct.

(B) Schematic of Brf2 domain organization.

See also Figures S1, S2, and S7 and Tables S1 and S2.

and TRNAU1 promoters at resolutions of 1.9 Å, 2.2 Å, and 2.7 Å, respectively (Figures 1A and S1A–S1D; Table S2). In all three cases, the double-stranded DNA scaffold used for crystallization was 28 bp long and corresponded to promoter sequences extending 10 bp upstream and downstream of the TATA box (Figure S1A). Notably, the DNA path was not perturbed by the crystalline environment and was influenced only by the specific interactions with TBP and Brf2. Where not stated otherwise, we focus on the analysis of the complex solved at the highest resolution, the Brf2-TBP/U6-2 complex (hereafter referred to as the Brf2-TBP/DNA complex), but the conclusions apply to all three complexes.

The overall architecture of the Brf2-TBP/DNA complex (Figure 1) is reminiscent of the TFIIB-TBP/DNA complex (Nikolov et al., 1995; Tsai and Sigler, 2000), thus providing experimental support to the proposed common architectural organization of TFIIB-related factors and TFIIB (Vannini and Cramer, 2012) (Figure S1B). In the Brf2-TBP/RPPH1 structure, the Brf2-TBP complex is bound to the DNA with inverse polarity, probably due to the perfect dyad symmetry of the TATA box at this promoter (Figures S1A and S1C).

Whether associated with Brf2 or TFIIB, TBP interacts with the TATA box in an undistinguishable manner, generating a virtually identical local bend in the DNA. However, as a result of specific Brf2/DNA interactions, the path of the DNA backbone deviates at the TATA flanking regions (Figure S1B). Modeling of a Pol III closed PIC using the Brf2-TBP/DNA complex revealed no clashes with the polymerase core and a DNA path that strongly resembles that of the human Pol II closed PIC (He et al., 2013) (Figure 2). In this model, the path of the DNA downstream of the TATA box points directly toward the Pol III subunits C39 and C62, consistent with their DNA binding activity and their functional role in DNA melting and open PIC stabilization (Brun et al., 1997; Lefèvre et al., 2011).

Brf2/DNA-Specific Interactions

Brf2 contacts extensively the phosphate backbone of the DNA and establishes sequence-specific contacts with both the upstream and downstream TATA flanking regions (Figures 1A and S1A–S1C). The Brf2 N-terminal cyclin repeat is structurally related to the corresponding domain in TFIIB, however in the Brf2-TBP/DNA complex the minor groove of the DNA is more intimately contacted via a helix-turn-helix motif (Figures 1A and 3A). The highly conserved dyad K113 and K114 (Figure S1E) contacts the phosphate backbone of the DNA on opposite sides, allowing the insertion of a short helix into the minor groove and the consequent direct recognition of bases A_{+3} and G'_{+4} (numbering is relative to the edge of the TATA box, with the non-template strand designated by a prime) (Figure 3A). The side chain of R110 forms a direct hydrogen bond with A_{+3} and a water-mediated hydrogen bond with $G'_{+4,}$ whereas the main chain carbonyl oxygen of A108 forms a direct hydrogen bond

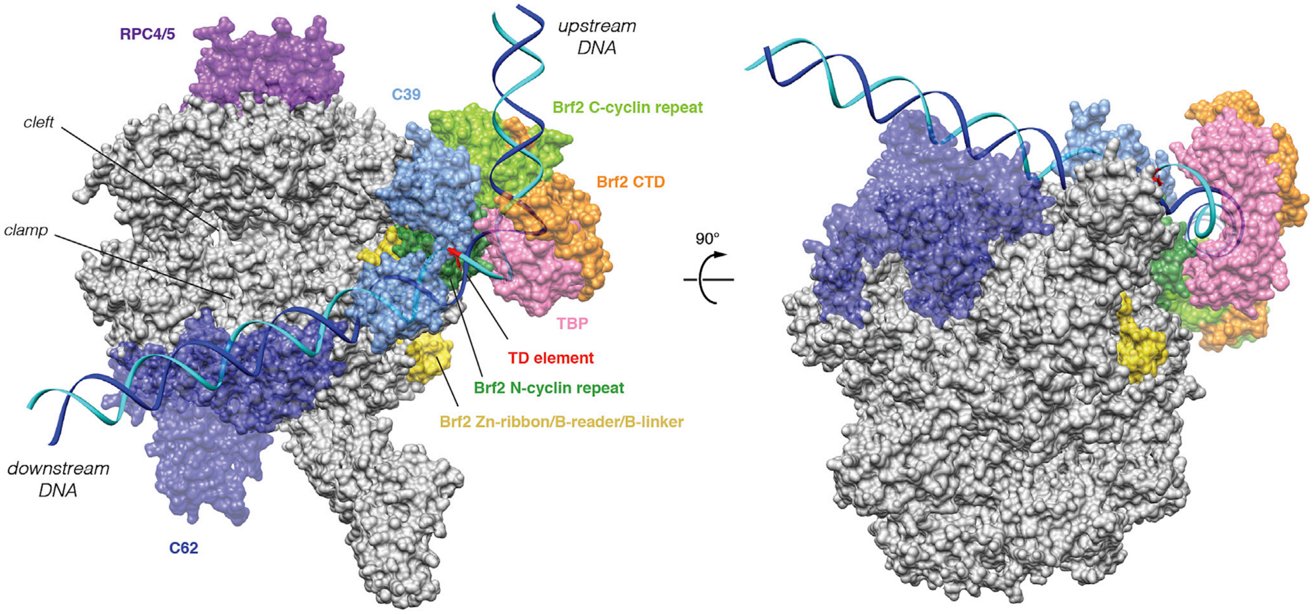

Figure 2. Architecture of the Human Pol III PIC

Model of a Pol III PIC (Vannini and Cramer, 2012) generated using the Brf2-TBP/DNA complex reveals that the path of the downstream DNA points toward the Pol III-specific subunits C39 and C62, and resembles the path observed in yeast and human Pol II PIC (He et al., 2013; Mühlbacher et al., 2014).
See also Figure S7.

with G'_{+4}. These interactions locally distort the DNA, leaving the base T'_{+3} unstacked at its downstream edge. A nucleobase T at position +3 on the non-template strand, followed by a nucleobase A, T, or G (collectively abbreviated as D) at position +4, is notably enriched at Brf2-dependent promoters (Figure S2A). We investigated the importance of the TD motif, which is also a conserved feature of the Pol II BRE_d (Deng and Roberts, 2005), on the formation of a Brf2-TBP/DNA ternary complex, using electrophoretic mobility shift assays (EMSAs) (Figure 3B). In agreement with the observed role of R110 in specific recognition of A_{+3}, a Brf2 R110A mutant displayed a reduced affinity for DNA compared to Brf2 wild-type, an effect that was most prominent in presence of an A nucleobase at position +3 of the template strand, suggesting a functional discrimination between T'_{+3} -containing and T'_{+3} -less promoters (Figures 3B and S2A). Thus, the conserved TD element, a dinucleotide step characterized by low unstacking energies (Protozanova et al., 2004), may represent a site at the downstream edge of the TATA box where DNA melting is favored as a result of the specific interactions with Brf2 R110 and A108. Indeed, in the Brf2-TBP/RPPH1 structure, in which the canonical TD motif is replaced with TC nucleobases, the $T'_{+3}C'_{+4}$ dinucleotide is still specifically recognized by R110 but with an altered set of interactions that leaves the nucleobase T'_{+3} unperturbed and regularly stacked at both edges, likely due to the higher stacking energy of this dinucleotide step (Figure S2C).

The Brf2 C-terminal cyclin repeat interacts with the major groove upstream of the TATA-box, similarly to TFIIB albeit more intimately (Lagrange et al., 1998; Tsai and Sigler, 2000) (Figures 1A and 3C). Brf2 residue Y260 establishes a hydrogen bond and a T-shaped π-π interaction with nucleobase C_{-4} and an additional T-shaped π-π interaction with nucleobase C_{-3} (Figure 3C). T-shaped π-π interactions are favored in the presence of pyrimidines (Wilson et al., 2014), explaining their enrichment, in particular for C, observed at −4 and −3 positions (Figure S2A). The hydrogen bond between Y260 and C_{-4} explains the presence of a C_{-4} nucleobase (complementary G in the non-template strand) at the vast majority of Brf2-dependent promoters, since a base-specific hydrogen bond is only possible with the amine group of a pyrimidine nucleobase C or purine nucleobase A at that position. Indeed, substitution of C_{-4} with a G or a T residue, or exchange of both adjacent pyrimidines with purines, resulted in reduced binding (Figure 3D). A nucleobase A at position −4 is well tolerated since it can establish an hydrogen bond with Y260, but exclusively in presence of a pyrimidine at position −3, in order to preserve the additional π-π interaction. In summary, the structural and functional data underscore the preference for a C nucleobase and a pyrimidine nucleobase (C or T) at positions −4 and −3, respectively, of the template strand. We named this Brf2-specific dinucleotide step the "GR" element (Figure S2A).

Modular Architecture of Brf2 CTD

The Brf2 CTD is organized into three conserved modular structural elements (Figures 1 and S1E). Brf2 conserved residues 291–314 fold into an unusual semi-circular α helix, which we named the "arch." Brf2 truncation at position D289, but not at positions R394, G380, G348, or A311, prevented the formation of a SNAPc-Brf2-TBP/DNA complex (Figure S3A), consistent with the arch constituting a main $SNAP_c$-binding interface.

The C-terminal part of the Brf2 CTD (residues 374–419) folds into a TBP "anchor domain," which structurally resembles Brf1

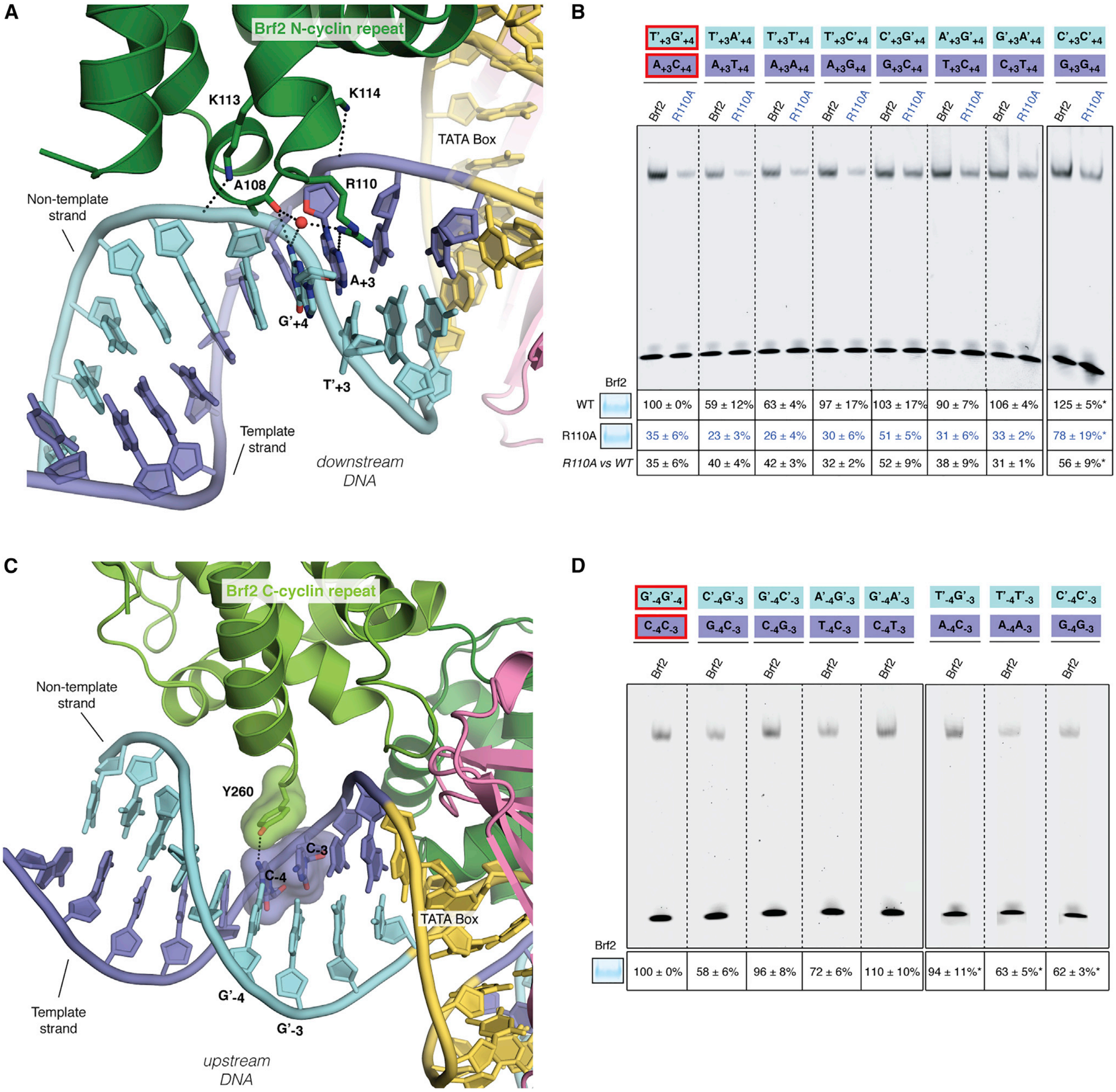

Figure 3. Brf2/DNA Sequence-Specific Interactions

(A) Close-up view of the TATA box (yellow), downstream flanking region and sequence-specific interactions with Brf2. DNA template and non-template strands are in blue and cyan respectively.

(B) Substitutions at positions +3 and +4 of the wild-type (circled in red) U6-2 promoter decrease binding of a R110A mutant, in particular when a T nucleobase is present at position +3 on the non-template strand (in cyan). R110A versus wild-type (WT) is the ratio between the percentage of binding of the mutant versus wild-type Brf2 proteins.

(C) Close-up view of the TATA box (yellow), upstream flanking region and sequence-specific interactions with Brf2. DNA template and non-template strands are in blue and cyan respectively.

(D) Substitutions at positions −3 and −4 of the wild-type (circled in red) U6-2 promoter reveal more efficient complex formation with a pyrimidine nucleobase and a C nucleobase at positions −3 and −4 of the template strand, respectively. (B and D) The intensity of the complex formed with TBP, U6-2 non mutated sequence and wild-type Brf2 (lane 1) was used as a reference for relative quantification. *Indicates samples that were quantified relative to a distinct wild-type sequence reference not shown on the figure. Representative gels of three independent experiments. The data shown are the mean values and SE of three independent experiments. In the insets, 10 μl of a typical binding reaction (25 μl total) with Brf2 wild-type or Brf2 mutants were loaded on a SDS-PAGE gel and stained with Coomassie-blue, confirming that equal amounts of protein of comparable quality were used for EMSA assays.

See also Figures S2 and S7.

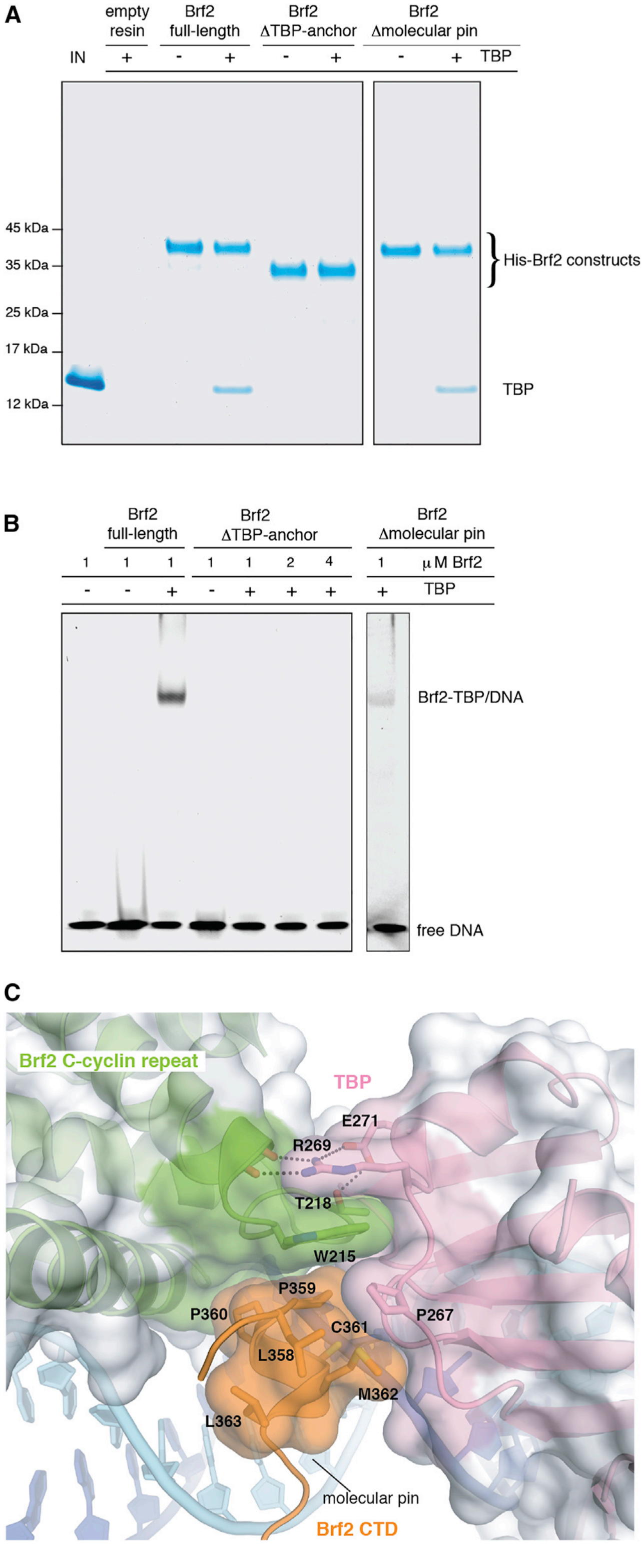

Figure 4. The Brf2 Molecular Pin

(A) The Brf2 TBP anchor domain but not the molecular pin is essential for Brf2-TBP interaction in absence of the DNA, as shown by a pull-down assay.

(B) The Brf2 TBP anchor domain and the molecular pin are essential for the formation of a Brf2-TBP/DNA complex, as shown in an EMSA.

(C) Close-up view of the Brf2 molecular pin at the interface between the Brf2 C-cyclin repeat, TBP, and the DNA.

See also Figures S3 and S7.

homology region II and binds TBP on its convex surface similarly to other TBP-associated factors (Anandapadamanaban et al., 2013; Juo et al., 2003) (Figure S3B). Remarkably, deletion of the TBP anchor domain (residues 365–419) not only abolishes the binding of Brf2 to TBP but also abrogates the formation of a ternary Brf2-TBP/DNA complex (Figures 4A and 4B).

A Brf2-specific short structured element (residues 357–363), the "molecular pin," encompasses a conserved LPPC motif and lies at the ternary interface between the Brf2 C-terminal cyclin repeat, TBP, and the DNA (Figure S1E and Figure 4C). This helical element virtually pins the ternary complex together, juxtaposing onto a hydrophobic pocket at the interface between TBP and the Brf2 C-terminal cyclin repeat, which, in contrast to what is observed for TFIIB, strongly interact together. The Brf2 residue W215 forms a hydrophobic stack with TBP residues R269 and P267 (Figure 4C) and is conserved throughout Brf2 evolution and in human and yeast Brf1, implying a conserved general architecture of Pol III Brf1- and Brf2-TBP complexes (Figures S1E and S2B). These additional strong interactions between the Brf2 and Brf1 C-terminal cyclin repeats and TBP may contribute to the observed increased stability of the Pol III-PIC, as compared to Pol II (Arimbasseri et al., 2013). The deletion of the molecular pin does not impair Brf2 binding to TBP in the absence of DNA but severely impairs the formation of a ternary complex, underscoring the central role of the molecular pin in the formation of a functional Brf2-TBP/DNA complex (Figures 4A and 4B). At the tip of the molecular pin, the Brf2 residue C361 is buried in the groove between two adjacent phosphate groups of the DNA backbone, establishing both van der Waals and water-mediated hydrogen bonds at the upstream edge of the TATA box (Figure 5A).

Brf2 Is Redox Regulated

A recurring theme in redox signaling by transcription factors is the presence of a reactive cysteine thiol that can cycle through reduced and oxidized states, "sensing" the redox environment of the cell (Brigelius-Flohe and Flohe, 2011). We noticed a remarkable structural similarity between the conserved Brf2 C361 and NF-κB p50 C59 (Ghosh et al., 1995) (Figure S3C), a DNA-binding cysteine residue regulated via oxidative modifications in vivo (Pineda-Molina et al., 2001), prompting us to investigate the redox properties of Brf2.

Using tandem mass-spectrometry, we could detect reversible oxidative modifications of C361 upon overnight incubation of Brf2 in absence of reducing agents and in presence of dimedone, a cyclic diketone that specifically reacts with sulfenic acid (Figure S4). The strictly conserved C361 and the nearby non-conserved C370 were the only two cysteine residues clearly identified with bound dimedone, suggesting that these residues reside in a local protein environment prone to oxidation. Indeed, the chemical environment surrounding these residues is enriched in positively charged residues (K363, K367, and R368),

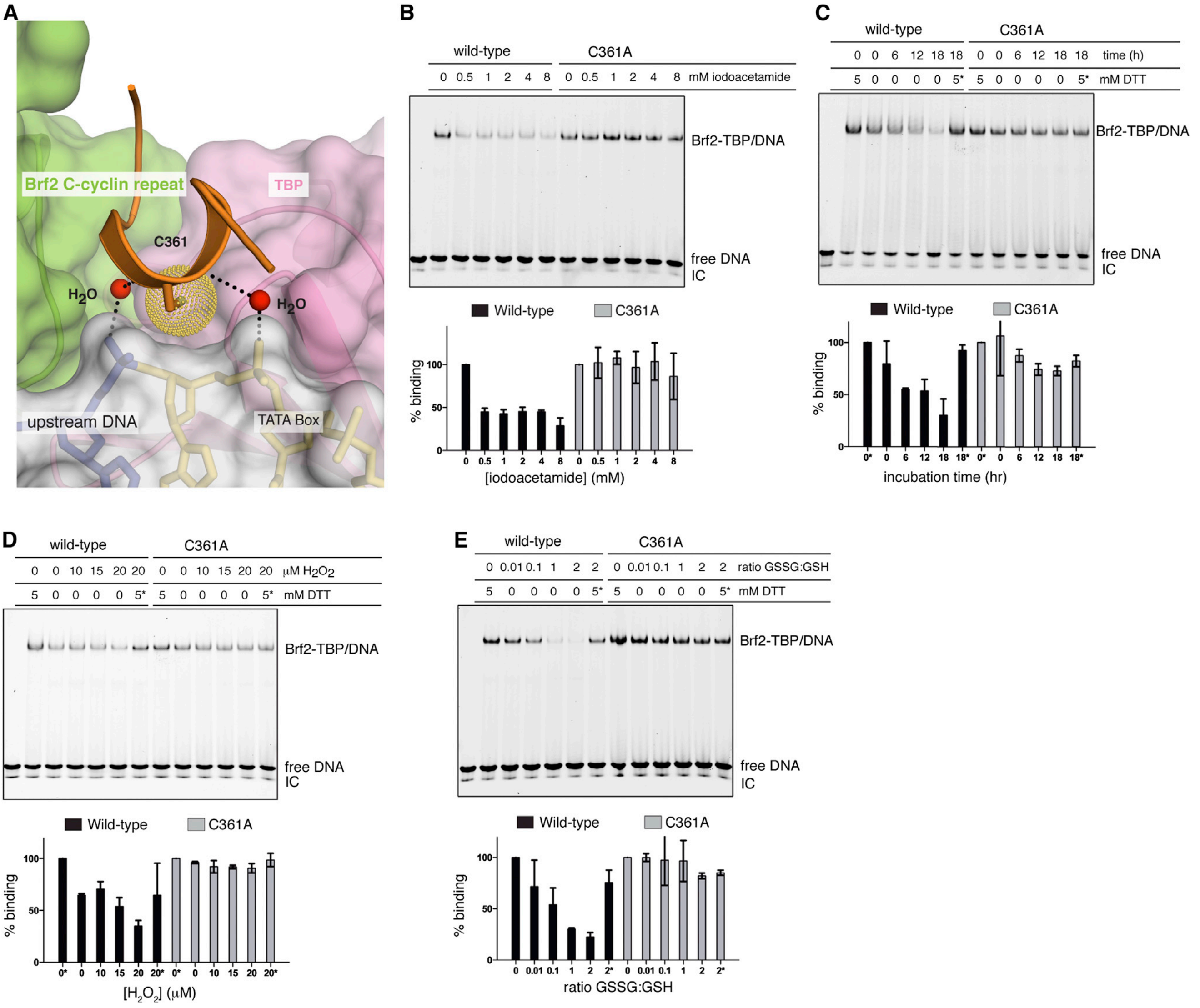

Figure 5. Brf2 Redox Regulation

(A) Close-up view of C361 at the ternary interface between the Brf2 C-cyclin repeat, TBP, and the upstream edge of the TATA box. Yellow dots represent the van der Waals radius of the sulfur atom.

(B) Representative EMSA of Brf2-TBP/DNA complexes upon pre-incubation of Brf2 proteins with the alkylating agent iodoacetamide. The IC band was used for loading normalization. *Indicates addition of the reducing agent after the oxidative treatment during complex assembly.

(C) Representative EMSA of Brf2-TBP/DNA complexes upon removal of reducing agent (DTT) and incubation over time. The IC band was used for loading normalization. *Indicates addition of the reducing agent after the oxidative treatment during complex assembly.

(D) Representative EMSA of Brf2-TBP/DNA complexes upon pre-incubation of Brf2 proteins with H_2O_2. The IC band was used for loading normalization. *Indicates addition of the reducing agent after the oxidative treatment during complex assembly.

(E) Representative EMSA of Brf2-TBP/DNA complexes upon pre-incubation of Brf2 proteins with gradients of oxidized/reduced glutathione (GSSG:GSH). The IC band was used for loading normalization. *Indicates addition of the reducing agent after the oxidative treatment during complex assembly.

See also Figures S3, S4, and S7.

which are known to stabilize thiolate anions, reducing the pK_a of the cysteine residues and thus resulting in a sulfur atom that is more susceptible to oxidation (Lo Conte and Carroll, 2013). Protein sulfenylation is a reversible post-translational modification that is emerging as a novel regulatory mechanism with particular relevance in redox signal transduction (Gupta and Carroll, 2014). In this context, the formation of mixed disulfide bonds between sulfenic acid intermediates and low molecular-weight thiols such as glutathione constitutes a common cellular mechanism to prevent progression toward irreversible oxidation states, thus ensuring reversible regulation. We confirmed by tandem mass-spectrometry that upon incubation with oxidized glutathione, Brf2 is efficiently *S*-glutathionylated specifically at residues C361 and C370 (Figure S4). Altogether, the mass-spectrometry

data indicate that Brf2 C361 and C370 are highly reactive cysteine residues that are prone to oxidation and can be *S*-glutathionylated in vitro.

We thus tested the functional relevance of Brf2 oxidative modifications on the formation of functional Brf2-TBP/DNA complexes using EMSAs. Incubation of Brf2 with iodoacetamide, a low molecular-weight compound that irreversibly alkylates reactive cysteine and lysine residues in proteins and peptides, resulted in a severe reduction of Brf2-TBP/DNA complex assembly (Figure 5B). This effect was mediated by residue C361, since a Brf2 C361A mutant was insensitive to treatments with the alkylating agent and displayed unaltered affinity for TBP/DNA complexes (Figure 5B and S3F). Thus, out of a total of 16 cysteine residues, Brf2 C361 is the sole reactive cysteine negatively regulating the formation of the Brf2-TBP/DNA ternary complex upon alkylation. Brf2 oxidation caused by removal of reducing agents and incubation over time impaired the formation of the ternary complex, an effect that was mostly reversible and mediated by C361 (Figure 5C). This finding suggests that oxidative modifications of C361 can reversibly modulate the assembly of the ternary complex and that a C361A mutation can confer redox-insensitivity to Brf2. In agreement, incubation of wild-type Brf2 with low concentrations of H_2O_2 also impaired the formation of the complex, while the C361A mutant remained insensitive (Figure 5D), suggesting that Brf2 C361 is susceptible to regulation by reactive oxygen species (ROS). Moreover, a Brf2 C361D mutant, structurally mimicking the oxidation state of C361 as a cysteic acid, was compromised in ternary complex formation with an apparent 50-fold reduction in affinity for TBP-DNA complexes, while still capable of efficiently binding to TBP (Figures S3D–S3F). To better simulate the perturbations of the redox potential that occur during oxidative stress in the cell, we treated Brf2 with gradients of oxidized/reduced glutathione (GSSG/GSH), whose ratio increases drastically during oxidative stress (Jones, 2006). The formation of the ternary complex was highly sensitive to Brf2 treatments with GSSG/GSH, an effect that was fully reversed by addition of reducing agents and again exclusively mediated by C361, since the C361A mutant was insensitive to treatments with high GSSG/GSH ratios (Figure 5E).

Collectively, these results support a redox-sensing functional role of Brf2 C361, whose critical localization at the Brf2-TBP/DNA ternary interface enables modulation of the assembly of a functional complex.

Brf2-Dependent Pol III Transcription Is Redox-Regulated in Living Cells

Having established Brf2 as a bona fide redox sensor, we asked whether synthesis of Brf2-dependent RNAs might be regulated in response to oxidative stress in living cells. We monitored the intracellular levels of Brf2-dependent transcripts by qRT-PCR in MRC5 lung fibroblast cells challenged by exposure to tert-butylhydroperoxide (t-BHP), a potent inducer of oxidative stress. Remarkably, all the Brf2-dependent transcripts tested were severely reduced upon treatment, while leucine tRNA precursors (p-tRNA), a Brf1-dependent transcript, remained unchanged (Figure 6A). We then focused on the selenocysteine (SeCys) tRNA, an essential tRNA encoded by a single active gene in mammalian genomes (James Faresse et al., 2012; Oler et al., 2010). Intracellular levels of SeCys p-tRNA were severely reduced in a t-BHP concentration- and exposure time-dependent manner (Figure 6B). Removal of the oxidative agent after an acute exposure promptly restored high levels of SeCys p-tRNA (Figure 6C). Importantly, oxidative stress-induced decrease of SeCys p-tRNA was Brf2-dependent, since transient transfection with Brf2 overexpression vectors rescued this effect (Figure 6D). Strikingly, overexpression of the Brf2 redox-insensitive C361A mutant led to an increase of SeCys p-tRNA, suggesting the loss of a negative regulatory step. Conversely, overexpression of the Brf2 oxidized-mimic C361D did not affect SeCys p-tRNA, suggesting that this mutant is virtually inactive (Figure 6D). In agreement with the existence of a post-translational regulatory mechanism, the decrease of SeCys p-tRNA levels observed upon treatment of MRC5 cells with t-BHP did not correlate with reduced Brf2 expression levels, which in fact increased during oxidative stress (Figure 7A). Furthermore, we observed an 80% reduction of SeCys mature tRNA (m-tRNA) levels in cells challenged with t-BHP, an effect that was reversed by overexpression of Brf2 (Figure S5A). Despite tRNAs generally being considered as molecules with a relatively long half-life, this result is in agreement with previously published data highlighting the higher rate of decay of SeCys tRNA when compared to other tRNAs (Jameson et al., 2002).

Redox Sensing by Brf2 Modulates Resistance to Oxidative Stress in Normal and Cancer Cells

SeCys tRNA is essential for the synthesis of selenoproteins, the vast majority of which are involved in ROS detoxification and in the maintenance of cellular redox homeostasis (Kasaikina et al., 2012). To test whether the observed Brf2-dependent reduction of SeCys tRNA levels upon oxidative stress can impact the synthesis of selenoproteins and, consequently, resistance to ROS, we monitored the expression levels of a group of selenoproteins (Gpx1, Gpx4, SelM, and Sep15) in which a SeCys residue is present toward the N terminus of the protein (Kasaikina et al., 2012). In conditions of oxidative stress, as monitored by the increased steady-state levels of the nuclear factor erythroid 2-related factor 2 (Nrf2), intracellular levels of all the selenoproteins tested were substantially reduced in MRC5 cells, an effect that was rescued by overexpression of Brf2, while no differences were observed in unchallenged cells (Figure 7A). Strikingly, Brf2 overexpression in MRC5 cells resulted in a marked acquired resistance toward oxidative stress enabling MRC5 cells to evade apoptosis (Figure 7B). Paralleling the redox-induced changes in SeCys p-tRNA synthesis, overexpression of the Brf2 redox-insensitive C361A mutant enabled a more pronounced resistance to oxidative stress when compared to Brf2 wild-type, while the Brf2 oxidized-mimic C361D was severely impaired in conferring resistance to apoptosis (Figure 7C). Since Brf2 has been recently discovered as a top-scoring candidate driver in breast carcinomas (Sanchez-Garcia et al., 2014), we additionally investigated the effects of Brf2 overexpression on selenoproteins expression levels and evasion of apoptosis in MCF10A cells, a mammary epithelium cell line with low expression of Brf2. We observed a strong

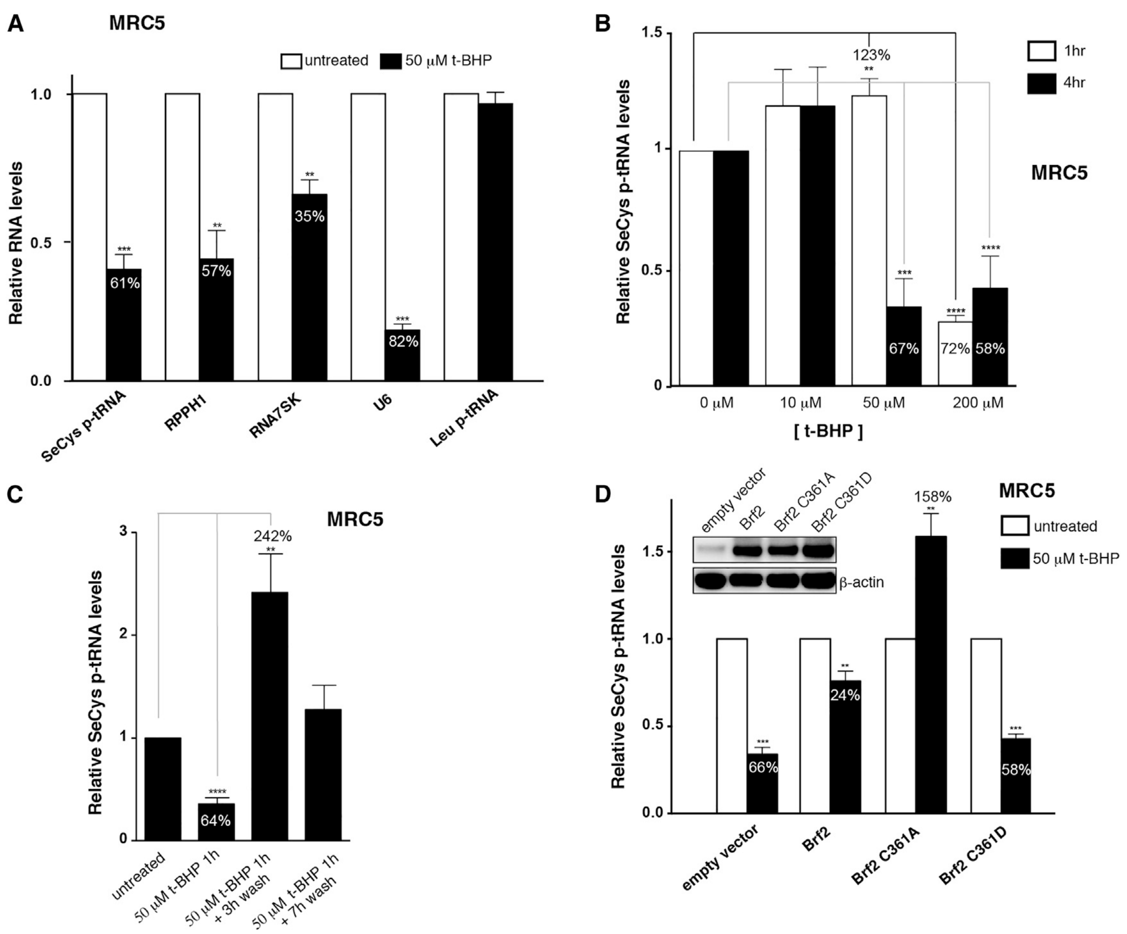

Figure 6. Brf2-Dependent Transcription Is Redox Regulated in Living Cells

(A) qRT-PCR analysis shows that Brf2-dependent-transcripts (SeCys p-tRNA, RPPH1, RNA7SK, and U6 snRNA) are globally downregulated during oxidative stress, while a Brf1-dependent transcript (Leu p-tRNA) remains unchanged.

(B) SeCys p-tRNA levels are strongly reduced in cells challenged with t-BHP relative to the unchallenged cells (as highlighted by gray and black lines, respectively) in a dose- and time-dependent manner, as measured by qRT-PCR.

(C) SeCys p-tRNA levels rapidly recover upon removal of the exogenous oxidative stress inducer, as measured by qRT-PCR. Wash indicates replacement of media containing t-BHP with fresh media.

(D) Effects of overexpression of Brf2 and Brf2 mutants (inset) on SeCys p-tRNA levels during oxidative stress, as measured by qRT-PCR.

The numbers indicated on the histograms represent the percentage of reduction of selenocysteine tRNA levels, while if numbers are indicated above the histograms they represent the percentage of increase. Cumulative data of at least three experiments, mean + SEM. Unpaired t test: *$p < 0.05$; **$p < 0.005$; ***$p < 0.0005$; ****$p < 0.0001$. $p > 0.05$ were deemed not significant and values were not reported.

See also Figures S5 and S7.

acquired resistance to oxidative stress that correlates with higher levels of selenoproteins, analogously to what observed in MRC5 cells (Figures S5B–S5D).

Conversely, to test the functional consequences of decreasing Brf2 activity during oxidative stress in cancer cells, we reduced the levels of Brf2 via small interfering RNA (siRNA) in A549 cells challenged with t-BHP. A549 are epithelial human lung adenocarcinoma cells displaying high Brf2 expression and a generally increased resistance to t-BHP treatment when compared to MRC5 fibroblasts (Figures 7A and 7D). In A549 cells, we could additionally monitor the expression levels of Gpx2, a selenoprotein upregulated by Nrf2 during oxidative stress (Brigelius-Flohé et al., 2012) and overexpressed in colorectal and prostate cancer (Emmink et al., 2014; Naiki et al., 2014). As for the primary cell lines, we observed an inverse correlation between cellular commitment to apoptosis and the expression level of selenoproteins (Figures 7A and 7D). Levels of all the selenoproteins tested, including Gpx2 that was upregulated during oxidative stress, were reduced in Brf2-silenced cells challenged with 50 μM t-BHP, a condition that induced apoptosis (Figures 7A and 7D). This effect, as well as the decrease of SeCys p-tRNA levels, was reversed by concomitant overexpression of a siRNA-resistant form of Brf2 (Figure S6), suggesting a direct involvement of Brf2 in the oxidative stress response pathway and in the acquired resistance to oxidative stress observed in human lung adenocarcinoma cells.

DISCUSSION

The structures of Brf2-TBP/DNA ternary complexes reveal a general conservation of the architecture of TFIIB-related factors and specific recognition of DNA elements by Brf2. A TD element at the downstream edge of the TATA box, a central component of the BRE_d Pol II core promoter element (Deng and Roberts, 2005), is specifically recognized by a Brf2 minor groove interacting element, resulting in a local distortion of the nucleic acid structure and a partially unstacked T nucleobase (Figures 2, 3A, and S2C), suggesting that BRE_d and BRE_d-like elements could represent sites primed for DNA melting. In this respect, the winged helix domains of yeast Pol III subunit C34 and Pol II transcription factor TFIIF (Tfg2 subunit), which have been involved in open complex formation and/or its stabilization (Brun et al., 1997; Mühlbacher et al., 2014), have been located exactly opposite of this site by cross-linking coupled with mass-spectrometry (Mühlbacher et al., 2014; Wu et al., 2012).

A completely unexpected finding was the discovery of a redox-sensing regulatory module embedded in a TFIIB-related core transcription factor, implying a direct redox-dependent control of a eukaryotic nuclear RNA polymerase. Fewer than 20 Brf2-dependent genes are actively transcribed in mammalian cells, but their products are all involved in key functions (Table S1). We have focused on the SeCys tRNA gene, since selenoproteins are directly involved in the oxidative stress response. We discovered that levels of SeCys tRNAs and selenoproteins are strongly reduced during oxidative stress in living cells in a Brf2-dependent manner, and this effect is inversely correlated with oxidative stress-induced apoptosis (Figures 6, 7, and S5).

Abrogating selenoprotein expression or expression of truncated selenoproteins induces apoptotic cellular death, sensitization toward oxidative stress and a reversion of the cancerous phenotype (Anestal et al., 2008; Emmink et al., 2014; Yoo et al., 2013). Reduced selenoprotein expression or generation of defective truncated selenoproteins might occur upon prolonged activation of the Nrf2 pathway, which upregulate the synthesis of TrxR1 and Gpx2 mRNAs, in conjunction with limited intracellular amounts of SeCys tRNAs. Indeed, we show that levels of Secys p-tRNAs, SeCys m-tRNAs and selenoproteins are reduced during prolonged oxidative stress in a Brf2-dependent manner (Figures 6A, 6B, 7A, S5A, and S5C). Thus, Brf2 redox-dependent regulation constitutes a cellular blockade capable of generating pro-apoptotic signals upon prolonged oxidative stress, by limiting the availability of SeCys tRNA (Figure S7).

Redox-dependent activation of Nrf2 is one of the principal events of the oxidative stress response pathway and is constitutively activated in squamous cell lung and breast carcinomas (Brigelius-Flohé et al., 2012; Cancer Genome Atlas Research Network, 2012; Sjöblom et al., 2006). Major targets upregulated by Nrf2 upon oxidative stress include TrxR1 and Gpx2, two essential selenoproteins involved in the maintenance of redox homeostasis and anti-oxidant defense, which are found overexpressed in several forms of cancers (Biaglow and Miller, 2005; Brigelius-Flohé et al., 2012; Emmink et al., 2014; Naiki et al., 2014). Thus, we hypothesized that overexpression of Brf2, which is also observed in many forms of cancer (Cabarcas and Schramm, 2011), is required to overcome the Brf2-dependent reduction of SeCys tRNAs observed during prolonged oxidative stress, in order to maintain sufficient expression levels of selenoproteins required for ROS detoxification and redox homeostasis. In agreement with this model, reducing Brf2 protein levels in A549 lung adenocarcinoma cells via siRNA resulted in diminished levels of selenoproteins (Figure 7A) and a considerable sensitization toward t-BHP, an inducer of oxidative stress (Figure 7D). This finding strongly supports a model of Brf2 as a key human redox-sensor involved in the oxidative stress pathway and mechanistically links its overexpression to malignancy, via a mechanism that enables cancer cells to evade apoptosis in conditions of prolonged oxidative stress, a hallmark of cancer (Hanahan and Weinberg, 2000). In this context, ectopic overexpression of Brf2 in lung fibroblasts (Figures 7A–7C) and mammary epithelial cells (Figure S5) reveals Brf2 oncogenic potential under oxidative stress conditions, supporting its role as an oncogenic driver in lung squamous cell carcinoma (Lockwood et al., 2010) and breast cancer (Sanchez-Garcia et al., 2014). Whether pro-apoptotic signals are generated exclusively in response to decreased intracellular levels of SeCys tRNA or whether additional Brf2-dependent transcripts are involved in the process remains to be determined.

The unexpected finding of Brf2 as a specialized Pol III TFIIB-related factor with redox-sensing properties suggests that the emergence and strict conservation of Brf2 in higher metazoans has been evolutionary driven to uncouple the transcriptional output of the Brf2-dependent promoters from the bulk of Pol III transcription, in order to operate a stringent redox-dependent control on a very small subset of Pol III genes. Since both selenoproteins and Brf2 are absent in lower eukaryotes such as plants and fungi, evolution of a redox-dependent transcription factor devoted to the transcription of SeCys tRNA must have represented an important event during evolution of higher complexity organisms.

EXPERIMENTAL PROCEDURES

A detailed description of protocols can be found in the Supplemental Experimental Procedures.

Protein Expression and Purification

Brf2 lacking the Zn-ribbon/B-reader/B-linker (62–419, N-terminal His-tagged) was co-expressed with a TBP-core construct (169–339) and used for structural determination. Full-length Brf2 (C-terminal His-tagged) was cloned into pSBET and used for biochemical assays. The Quickchange Site-Directed Mutagenesis kit (Agilent Technologies) was used to generate the Brf2 point mutants.

Crystallization, Data Collection, Structure Solution, and Refinement

Complexes were assembled at a final concentration of 60 μM and crystals were grown by mixing 1 μl each of complexes and crystallization solution (10%–20% PEG 3350, 50–100 mM $MgCl_2$, 2 mM DTT) in hanging drop plates. Following harvesting and cryo-cooling, diffraction data were collected at the Diamond Light Source (UK) and ESRF (France) synchrotrons. The structure was solved by molecular replacement, using TFIIB-TBP/DNA (Protein Data Bank: 1C9B) as the search model.

EMSAs

EMSAs where performed with 5′-Cy5 fluorescently labeled oligonucleotides. The gels were scanned with a Typhoon FLA9500 (GE Healthcare).

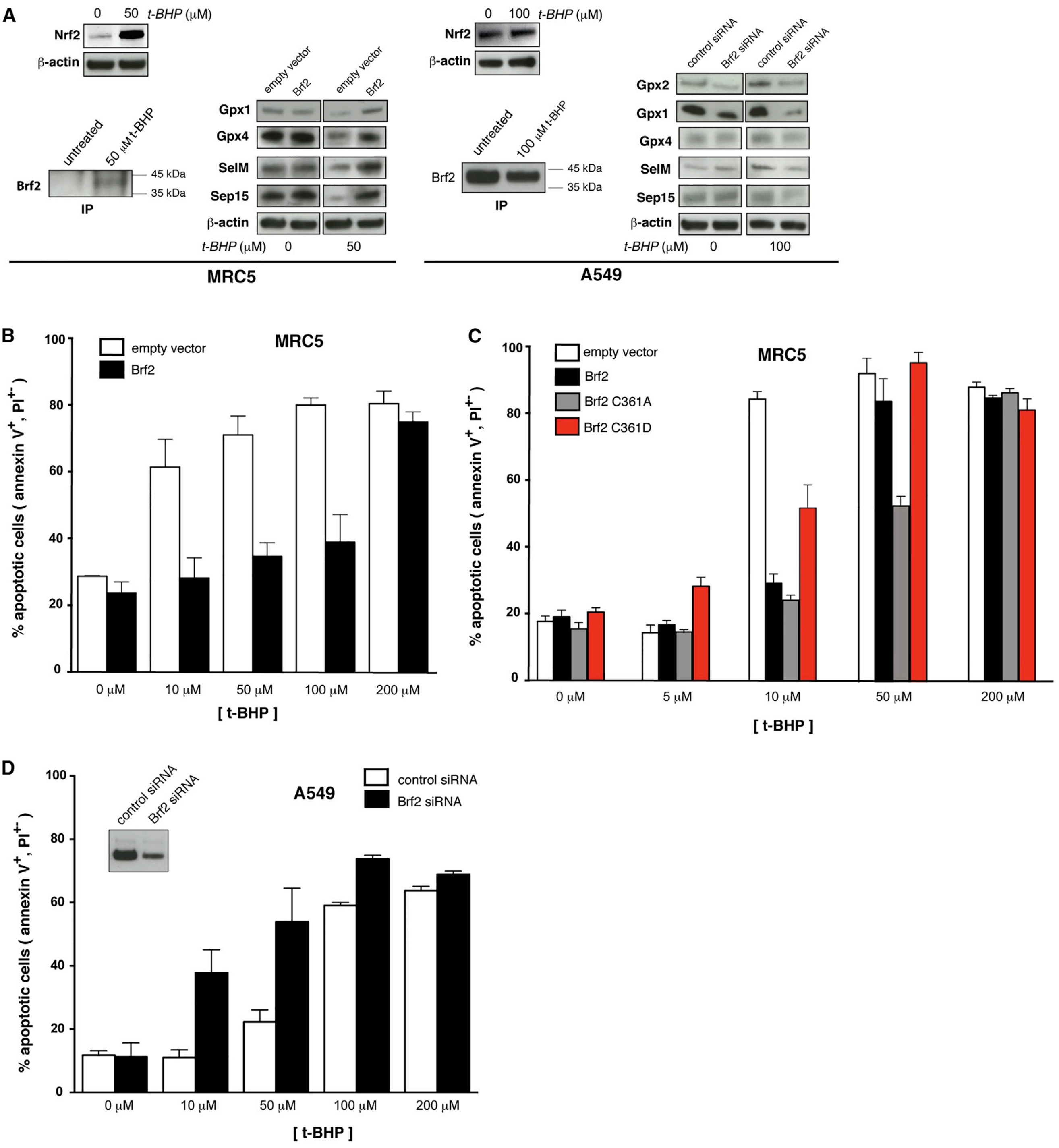

Figure 7. Selenoproteins Levels and Resistance to Oxidative Stress Are Regulated in a Brf2-Dependent Manner

(A) Manipulation of Brf2 protein levels affects selenoproteins expression levels during oxidative stress in MRC5 and A549 cells. In the upper insets, a western blot analysis of Nrf2 confirms induction of oxidative stress with 50 μM and 100 μM t-BHP in MRC5 and A549 cells, respectively. A western blot analysis of Brf2 immunoprecipitation from 10^7 MRC5 or A549 cells is shown in the lower insets (IP).

(B) Overexpression of Brf2 in MRC5 cells challenged with t-BHP results in decreased apoptosis as measured by FACS analysis via annexin V-FITC/PI staining. The y axis represents the % of apoptotic cells, including both cells in early (annexin V-positive and PI-negative) and late (annexin V-positive and PI-positive) apoptosis.

(C) Effects of overexpression of Brf2 wild-type and mutants on acquired resistance to apoptosis in MRC5 cells as measured by FACS analysis via annexin V-FITC/PI staining. The y axis represents the % of apoptotic cells, including both cells in early (annexin V-positive and PI-negative) and late (annexin V-positive and PI-positive) apoptosis.

(legend continued on next page)

Brf2 Pull-Down Experiments

Pull-Down experiments were performed by incubation of bait and prey proteins in binding buffer and loaded onto a His SpinTrap columns (GE Healthcare). Following washing and elution, samples were analyzed by SDS-PAGE.

Mass Spectrometry

Full-length Brf2 samples were digested with trypsin and infused into an LTQ Velos Orbitrap mass spectrometer (Thermo Fisher Scientific) to characterize cysteine oxidation states.

Fluorescence Polarization Assay

Binding of 5′-Alexa488-labeled oligonucleotides were monitored at different Brf2-TBP concentrations by fluorescence anisotropy at 25°C on a POLARstar Omega plate reader (BMG Labtech).

Immunopurification

Immunopurification from cells lysed in RIPA buffer were carried out using a chip-grade Brf2 antibody (ab17011, Abcam) covalently coupled to epoxy-magnetic beads (Life Technologies), according to the manufacturer's protocol.

Brf2 Overexpression and siRNA

Cells were transfected with 1.5 μg of pCDNA3.1 (empty vector control), Brf2WT-pCDNA3.1, Brf2C361A-pCDNA3.1, or Brf2C361D-pCDNA3.1 DNA in a 6-well plate format, with Lipofectamine 2000 according to manufacturer's instructions. In Figures 7C and S5D, cells were transfected with 1.5 μg of pCDNA3.1 (empty vector control) or 1.2 μg pCDNA3.1+ 0.3 μg of the relevant Brf2 construct.

For Brf2 knockdown, cells were transfected with siGENOME Human Brf2 siRNA (M-013340-00-0005, Dharmacon) in Figures 7A and 7D, and with siGENONE Human Brf2 siRNA (1) (D-013340-03-0010, Dharmcacon) or siGENOME Human Brf2 siRNA (2) (D-013340-04-0010, Dharmacon) in Figure S6, using Lipofectamine 2000 (Life Technologies). Allstars negative control (QIAGEN) was used for all control siRNA experiments.

qRT-PCR

Total RNA was extracted from treated cells with TRIzol reagent (Life Technologies) according to the manufacturer's instructions. SeCys p-tRNA was quantified using the relative standard curve method and the 5S rRNA as an endogenous control. SeCys m-tRNA was monitored using a previously published protocol (Honda et al., 2015) with minor modifications.

Flow Cytometry

Cell viability and apoptosis were assessed by flow cytometry using annexin V and propidium iodide staining.

ACCESSION NUMBERS

The accession numbers for the atomic coordinates and structure factors of the Brf2-TBP/U6-2, Brf2-TBP/TRNAU1, and Brf2-TBP/RPPH1 structures reported in this paper are Protein Data Bank: 4ROC, 4ROD, and 4ROE, respectively.

SUPPLEMENTAL INFORMATION

Supplemental Information includes Supplemental Experimental Procedures, seven figures, and two tables and can be found with this article online at http://dx.doi.org/10.1016/j.cell.2015.11.005.

AUTHOR CONTRIBUTIONS

J.G. carried out purification of protein/nucleic acid complexes, performed biochemical experiments, including EMSAs, fluorescence polarization and IPs, prepared Brf2-TBP/DNA crystals, collected crystallographic data, and solved the Brf2-TBP/DNA structures. K.S. originally cloned Brf2, carried out cellular work, and performed qRT-PCR and FACS analysis. J.G. and K.S. generated Brf2 mutants. N.G. preliminary expressed and purified Brf2. M.W. and A.J.T. performed mass spectrometry analysis. P.C., O.D., and N.H. generated $SNAP_c$-binding-deficient Brf2 mutants and performed EMSAs in presence of $SNAP_c$. A.V. designed and supervised research and wrote the manuscript with contributions from all authors.

ACKNOWLEDGMENTS

We thank N. Cronin at The Institute of Cancer Research (ICR) for help with the crystallization setup and data collection. We thank the staff at beamlines ID29 and ID23eh1 of the European Synchrotron Radiation Facility (France) and at beamlines I24 and IO3 of Diamond Light Source (UK) for help with data collection. We thank the laboratories of Chris Lord and Pascal Meier for help with siRNA and FACS analysis, Tracy Berg for help with qRT-PCR, and Fredrik Wallberg of the ICR Flow Cytometry Facility and Sarah Hanrahan of the Proteomics Core Facility. We thank Richard Maraia and Aneeshkumar Arimbasseri for critical reading of an early version of the manuscript and Sonja Christoph for support throughout the project. P.C., O.D., and N.H. were supported by the University of Lausanne and by Swiss National Science Foundation (SNSF) grant 31003A_132958. A.V. acknowledges the support of the Career Development Faculty Program of the ICR. Part of this work was supported by a Biotechnology and Biological Sciences Research Council (BBSRC) new-investigator award (BB/K014390/1) to A.V.

Received: August 21, 2015
Revised: October 14, 2015
Accepted: October 23, 2015
Published: December 3, 2015

REFERENCES

Anandapadamanaban, M., Andresen, C., Helander, S., Ohyama, Y., Siponen, M.I., Lundström, P., Kokubo, T., Ikura, M., Moche, M., and Sunnerhagen, M. (2013). High-resolution structure of TBP with TAF1 reveals anchoring patterns in transcriptional regulation. Nat. Struct. Mol. Biol. *20*, 1008–1014.

Anestal, K., Prast-Nielsen, S., Cenas, N., and Arner, E.S. (2008). Cell death by SecTRAPs: thioredoxin reductase as a prooxidant killer of cells. PloS ONE *3*, e1846.

Arimbasseri, A.G., Rijal, K., and Maraia, R.J. (2013). Comparative overview of RNA polymerase II and III transcription cycles, with focus on RNA polymerase III termination and reinitiation. Transcription *5*, e27639.

Biaglow, J.E., and Miller, R.A. (2005). The thioredoxin reductase/thioredoxin system: novel redox targets for cancer therapy. Cancer Biol. Ther. *4*, 6–13.

Brigelius-Flohe, R., and Flohe, L. (2011). Basic principles and emerging concepts in the redox control of transcription factors. Antioxid. Redox. Signal. *15*, 2335–2381.

Brigelius-Flohé, R., Müller, M., Lippmann, D., and Kipp, A.P. (2012). The yin and yang of nrf2-regulated selenoproteins in carcinogenesis. Int. J. Cell Biol. *2012*, 486147.

Brun, I., Sentenac, A., and Werner, M. (1997). Dual role of the C34 subunit of RNA polymerase III in transcription initiation. EMBO J. *16*, 5730–5741.

(D) Lowering Brf2 protein levels by siRNA in A549 cells challenged with t-BHP results in an increased cellular commitment to apoptosis as measured by FACS analysis via annexin V-FITC/PI staining. Inset: a western blot analysis of Brf2 immunoprecipitation from 10^7 A549 cells shows siRNA-induced Brf2 protein level reduction. The y axis represents the % of apoptotic cells, including both cells in early (annexin V-positive and PI-negative) and late (annexin V-positive and PI-positive) apoptosis.
See also Figures S5 and S7.

Cabarcas, S., and Schramm, L. (2011). RNA polymerase III transcription in cancer: the BRF2 connection. Mol. Cancer *10*, 47.

Cabart, P., and Murphy, S. (2001). BRFU, a TFIIB-like factor, is directly recruited to the TATA-box of polymerase III small nuclear RNA gene promoters through its interaction with TATA-binding protein. J. Biol. Chem. *276*, 43056–43064.

Cancer Genome Atlas Research Network (2012). Comprehensive genomic characterization of squamous cell lung cancers. Nature *489*, 519–525.

Carrière, L., Graziani, S., Alibert, O., Ghavi-Helm, Y., Boussouar, F., Humbertclaude, H., Jounier, S., Aude, J.-C., Keime, C., Murvai, J., et al. (2012). Genomic binding of Pol III transcription machinery and relationship with TFIIS transcription factor distribution in mouse embryonic stem cells. Nucleic Acids Res. *40*, 270–283.

Deng, W., and Roberts, S.G.E. (2005). A core promoter element downstream of the TATA box that is recognized by TFIIB. Genes Dev. *19*, 2418–2423.

Emmink, B.L., Laoukili, J., Kipp, A.P., Koster, J., Govaert, K.M., Fatrai, S., Verheem, A., Steller, E.J., Brigelius-Flohé, R., Jimenez, C.R., et al. (2014). GPx2 suppression of H2O2 stress links the formation of differentiated tumor mass to metastatic capacity in colorectal cancer. Cancer Res. *74*, 6717–6730.

Ghosh, G., van Duyne, G., Ghosh, S., and Sigler, P.B. (1995). Structure of NF-kappa B p50 homodimer bound to a kappa B site. Nature *373*, 303–310.

Gupta, V., and Carroll, K.S. (2014). Sulfenic acid chemistry, detection and cellular lifetime. Biochim. Biophys. Acta *1840*, 847–875.

Hanahan, D., and Weinberg, R.A. (2000). The hallmarks of cancer. Cell *100*, 57–70.

He, Y., Fang, J., Taatjes, D.J., and Nogales, E. (2013). Structural visualization of key steps in human transcription initiation. Nature *495*, 481–486.

Henry, R.W., Sadowski, C.L., Kobayashi, R., and Hernandez, N. (1995). A TBP-TAF complex required for transcription of human snRNA genes by RNA polymerase II and III. Nature *374*, 653–656.

Honda, S., Shigematsu, M., Morichika, K., Telonis, A.G., and Kirino, Y. (2015). Four-leaf clover qRT-PCR: A convenient method for selective quantification of mature tRNA. RNA Biol. *12*, 501–508.

James Faresse, N., Canella, D., Praz, V., Michaud, J., Romascano, D., and Hernandez, N. (2012). Genomic study of RNA polymerase II and III SNAPc-bound promoters reveals a gene transcribed by both enzymes and a broad use of common activators. PLoS Genet. *8*, e1003028.

Jameson, R.R., Carlson, B.A., Butz, M., Esser, K., Hatfield, D.L., and Diamond, A.M. (2002). Selenium influences the turnover of selenocysteine tRNA([Ser]Sec) in Chinese hamster ovary cells. J. Nutr. *132*, 1830–1835.

Jones, D.P. (2006). Redefining oxidative stress. Antioxid. Redox. Signal. *8*, 1865–1879.

Juo, Z.S., Kassavetis, G.A., Wang, J., Geiduschek, E.P., and Sigler, P.B. (2003). Crystal structure of a transcription factor IIIB core interface ternary complex. Nature *422*, 534–539.

Kasaikina, M.V., Hatfield, D.L., and Gladyshev, V.N. (2012). Understanding selenoprotein function and regulation through the use of rodent models. Biochim. Biophys. Acta *1823*, 1633–1642.

Kassavetis, G.A., Joazeiro, C.A., Pisano, M., Geiduschek, E.P., Colbert, T., Hahn, S., and Blanco, J.A. (1992). The role of the TATA-binding protein in the assembly and function of the multisubunit yeast RNA polymerase III transcription factor, TFIIIB. Cell *71*, 1055–1064.

Kassavetis, G.A., Nguyen, S.T., Kobayashi, R., Kumar, A., Geiduschek, E.P., and Pisano, M. (1995). Cloning, expression, and function of TFC5, the gene encoding the B'' component of the Saccharomyces cerevisiae RNA polymerase III transcription factor TFIIIB. Proc. Natl. Acad. Sci. USA *92*, 9786–9790.

Kassavetis, G.A., Letts, G.A., and Geiduschek, E.P. (1999). A minimal RNA polymerase III transcription system. EMBO J. *18*, 5042–5051.

Knutson, B.A., and Hahn, S. (2011). Yeast Rrn7 and human TAF1B are TFIIB-related RNA polymerase I general transcription factors. Science *333*, 1637–1640.

Lagrange, T., Kapanidis, A.N., Tang, H., Reinberg, D., and Ebright, R.H. (1998). New core promoter element in RNA polymerase II-dependent transcription: sequence-specific DNA binding by transcription factor IIB. Genes Dev. *12*, 34–44.

Lefèvre, S., Dumay-Odelot, H., El-Ayoubi, L., Budd, A., Legrand, P., Pinaud, N., Teichmann, M., and Fribourg, S. (2011). Structure-function analysis of hRPC62 provides insights into RNA polymerase III transcription initiation. Nat. Struct. Mol. Biol. *18*, 352–358.

Lo Conte, M., and Carroll, K.S. (2013). The redox biochemistry of protein sulfenylation and sulfinylation. J. Biol. Chem. *288*, 26480–26488.

Lobo, S.M., Tanaka, M., Sullivan, M.L., and Hernandez, N. (1992). A TBP complex essential for transcription from TATA-less but not TATA-containing RNA polymerase III promoters is part of the TFIIIB fraction. Cell *71*, 1029–1040.

Lockwood, W.W., Chari, R., Coe, B.P., Thu, K.L., Garnis, C., Malloff, C.A., Campbell, J., Williams, A.C., Hwang, D., Zhu, C.-Q., et al. (2010). Integrative genomic analyses identify BRF2 as a novel lineage-specific oncogene in lung squamous cell carcinoma. PLoS Med. *7*, e1000315.

López-De-León, A., Librizzi, M., Puglia, K., and Willis, I.M. (1992). PCF4 encodes an RNA polymerase III transcription factor with homology to TFIIB. Cell *71*, 211–220.

Lu, M., Tian, H., Yue, W., Li, L., Li, S., Qi, L., Hu, W., Gao, C., and Si, L. (2013). Overexpression of TFIIB-related factor 2 is significantly correlated with tumor angiogenesis and poor survival in patients with esophageal squamous cell cancer. Med. Oncol. *30*, 553.

Lu, M., Tian, H., Yue, W., Li, L., Li, S., Qi, L., Hu, W., Gao, C., and Si, L. (2014). TFIIB-related factor 2 over expression is a prognosis marker for early-stage non-small cell lung cancer correlated with tumor angiogenesis. PLoS ONE *9*, e88032.

Mühlbacher, W., Sainsbury, S., Hemann, M., Hantsche, M., Neyer, S., Herzog, F., and Cramer, P. (2014). Conserved architecture of the core RNA polymerase II initiation complex. Nat. Commun. *5*, 4310.

Naidu, S., Friedrich, J.K., Russell, J., and Zomerdijk, J.C.B.M. (2011). TAF1B is a TFIIB-like component of the basal transcription machinery for RNA polymerase I. Science *333*, 1640–1642.

Naiki, T., Naiki-Ito, A., Asamoto, M., Kawai, N., Tozawa, K., Etani, T., Sato, S., Suzuki, S., Shirai, T., Kohri, K., and Takahashi, S. (2014). GPX2 overexpression is involved in cell proliferation and prognosis of castration-resistant prostate cancer. Carcinogenesis *35*, 1962–1967.

Nikolov, D.B., Chen, H., Halay, E.D., Usheva, A.A., Hisatake, K., Lee, D.K., Roeder, R.G., and Burley, S.K. (1995). Crystal structure of a TFIIB-TBP-TATA-element ternary complex. Nature *377*, 119–128.

Oler, A.J., Alla, R.K., Roberts, D.N., Wong, A., Hollenhorst, P.C., Chandler, K.J., Cassiday, P.A., Nelson, C.A., Hagedorn, C.H., Graves, B.J., and Cairns, B.R. (2010). Human RNA polymerase III transcriptomes and relationships to Pol II promoter chromatin and enhancer-binding factors. Nat. Struct. Mol. Biol. *17*, 620–628.

Pineda-Molina, E., Klatt, P., Vázquez, J., Marina, A., García de Lacoba, M., Pérez-Sala, D., and Lamas, S. (2001). Glutathionylation of the p50 subunit of NF-kappaB: a mechanism for redox-induced inhibition of DNA binding. Biochemistry *40*, 14134–14142.

Protozanova, E., Yakovchuk, P., and Frank-Kamenetskii, M.D. (2004). Stacked-unstacked equilibrium at the nick site of DNA. J. Mol. Biol. *342*, 775–785.

Sanchez-Garcia, F., Villagrasa, P., Matsui, J., Kotliar, D., Castro, V., Akavia, U.D., Chen, B.J., Saucedo-Cuevas, L., Rodriguez Barrueco, R., Llobet-Navas, D., et al. (2014). Integration of genomic data enables selective discovery of breast cancer drivers. Cell *159*, 1461–1475.

Saxena, A., Ma, B., Schramm, L., and Hernandez, N. (2005). Structure-function analysis of the human TFIIB-related factor II protein reveals an essential role for the C-terminal domain in RNA polymerase III transcription. Mol. Cell. Biol. *25*, 9406–9418.

Schramm, L., and Hernandez, N. (2002). Recruitment of RNA polymerase III to its target promoters. Genes Dev. *16*, 2593–2620.

Schramm, L., Pendergrast, P.S., Sun, Y., and Hernandez, N. (2000). Different human TFIIIB activities direct RNA polymerase III transcription from TATA-containing and TATA-less promoters. Genes Dev. *14*, 2650–2663.

Sjöblom, T., Jones, S., Wood, L.D., Parsons, D.W., Lin, J., Barber, T.D., Mandelker, D., Leary, R.J., Ptak, J., Silliman, N., et al. (2006). The consensus coding sequences of human breast and colorectal cancers. Science *314*, 268–274.

Teichmann, M., Wang, Z., and Roeder, R.G. (2000). A stable complex of a novel transcription factor IIB- related factor, human TFIIIB50, and associated proteins mediate selective transcription by RNA polymerase III of genes with upstream promoter elements. Proc. Natl. Acad. Sci. USA *97*, 14200–14205.

Tsai, F.T., and Sigler, P.B. (2000). Structural basis of preinitiation complex assembly on human pol II promoters. EMBO J. *19*, 25–36.

Vannini, A., and Cramer, P. (2012). Conservation between the RNA polymerase I, II, and III transcription initiation machineries. Mol. Cell *45*, 439–446.

Wang, Z., and Roeder, R.G. (1995). Structure and function of a human transcription factor TFIIIB subunit that is evolutionarily conserved and contains both TFIIB- and high-mobility-group protein 2-related domains. Proc. Natl. Acad. Sci. USA *92*, 7026–7030.

White, R.J. (2011). Transcription by RNA polymerase III: more complex than we thought. Nat. Rev. Genet. *12*, 459–463.

Wilson, K.A., Kellie, J.L., and Wetmore, S.D. (2014). DNA-protein π-interactions in nature: abundance, structure, composition and strength of contacts between aromatic amino acids and DNA nucleobases or deoxyribose sugar. Nucleic Acids Res. *42*, 6726–6741.

Wu, C.-C., Herzog, F., Jennebach, S., Lin, Y.-C., Pai, C.-Y., Aebersold, R., Cramer, P., and Chen, H.-T. (2012). RNA polymerase III subunit architecture and implications for open promoter complex formation. Proc. Natl. Acad. Sci. USA *109*, 19232–19237.

Yoo, M.-H., Carlson, B.A., Gladyshev, V.N., and Hatfield, D.L. (2013). Abrogated thioredoxin system causes increased sensitivity to TNF-α-induced apoptosis via enrichment of p-ERK 1/2 in the nucleus. PLoS ONE *8*, e71427.

Supplemental Figures

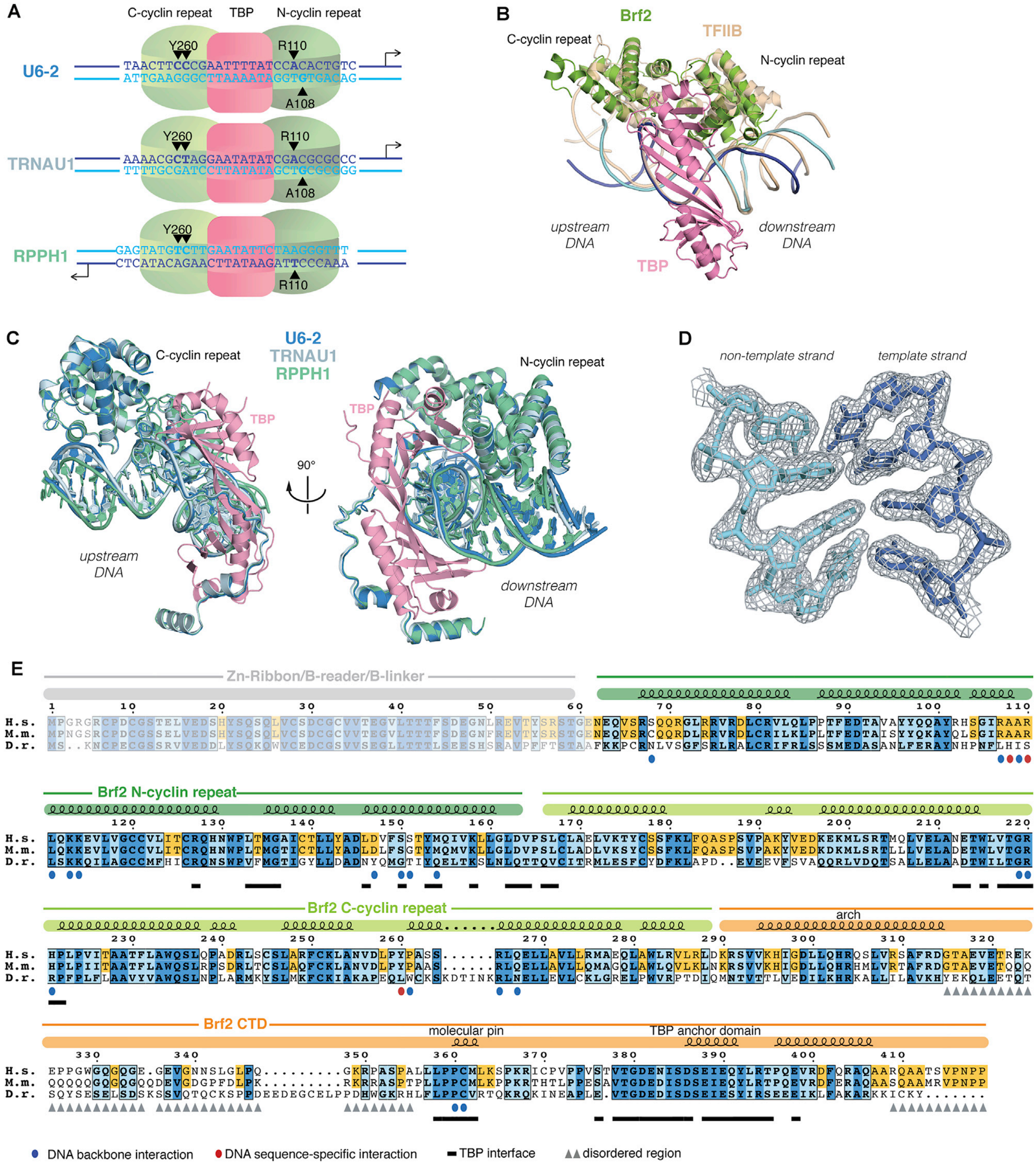

Figure S1. General Conservation of the Architecture of TFIIB and TFIIB-like Factors, Related to Figure 1

(A) Schematic of the architecture of the Brf2-TBP/DNA complexes and sequences of the DNA scaffold used for crystallization.

(B) Superimposition of Brf2-TBP/DNA and TFIIB-TBP/DNA (PDB: 1C9B). The structures were superimposed using TBP as a template for structural alignment.

(C) Superimposition of the Brf2-TBP/U6-2 (blue), Brf2-TBP/TRNAU1 (grey) and Brf2-TBP/RPPH1 (green) structures. The structures were superimposed using TBP as a template for structural alignment.

(D) Final electron density contoured at 1.2 s surrounding a tract of double-stranded DNA.

(E) Brf2 sequence conservation and domain organization of Homo sapiens (H.s.) Mus musculus (M.m.), and Danio rerio (D.r.).

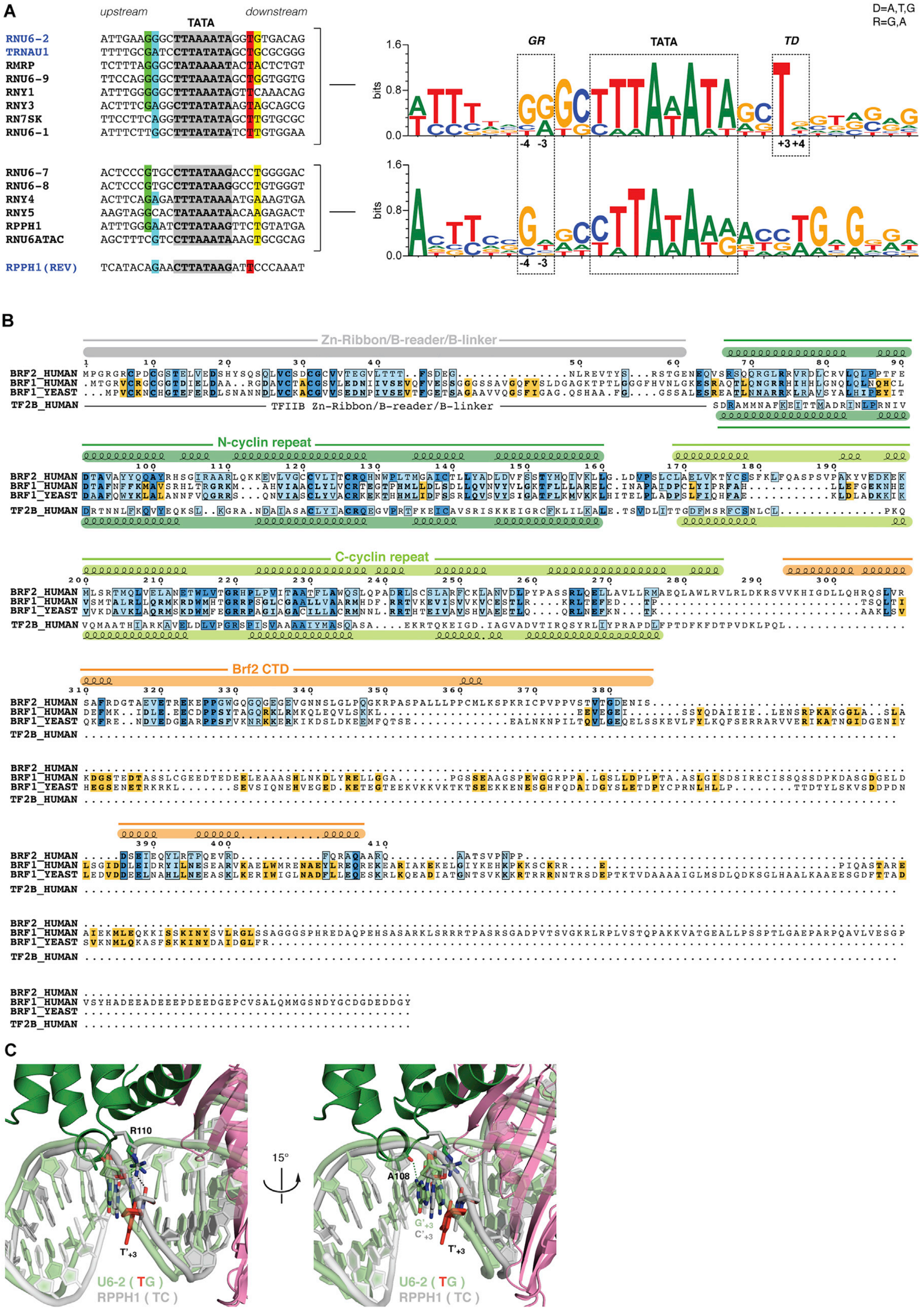

(legend on next page)

Figure S2. Sequence Alignments of Brf2 Promoters and Protein, Related to Figures 1 and 3

(A) Brf2-dependent promoters (40 nucleobase long centered around putative TATA boxes) were aligned using MEME (Bailey et al., 2009) by searching for a 16 nucleobase long consensus motif.

(B) Sequence and domain conservation between Brf2, Brf1 and TFIIB. Color-coding is as in Fig. 1. Brf2-Brf1 alignments are based on sequence conservation, while Brf2-TFIIB is based on a structural alignment.

(C) Two views of the specific interaction of Brf2 R110 and A108 with a TG (U6-2 in pale green) and TC (RPPH1 in light grey) dinucleotide step. In presence of the TG sequence, the T on the nontemplate strand (in red) is left unstacked at its downstream edge. In presence of a TC sequence, no local distortions of the DNA are observed. The two structures were superimposed by structurally aligning the Brf2 N-terminal cyclin repeats.

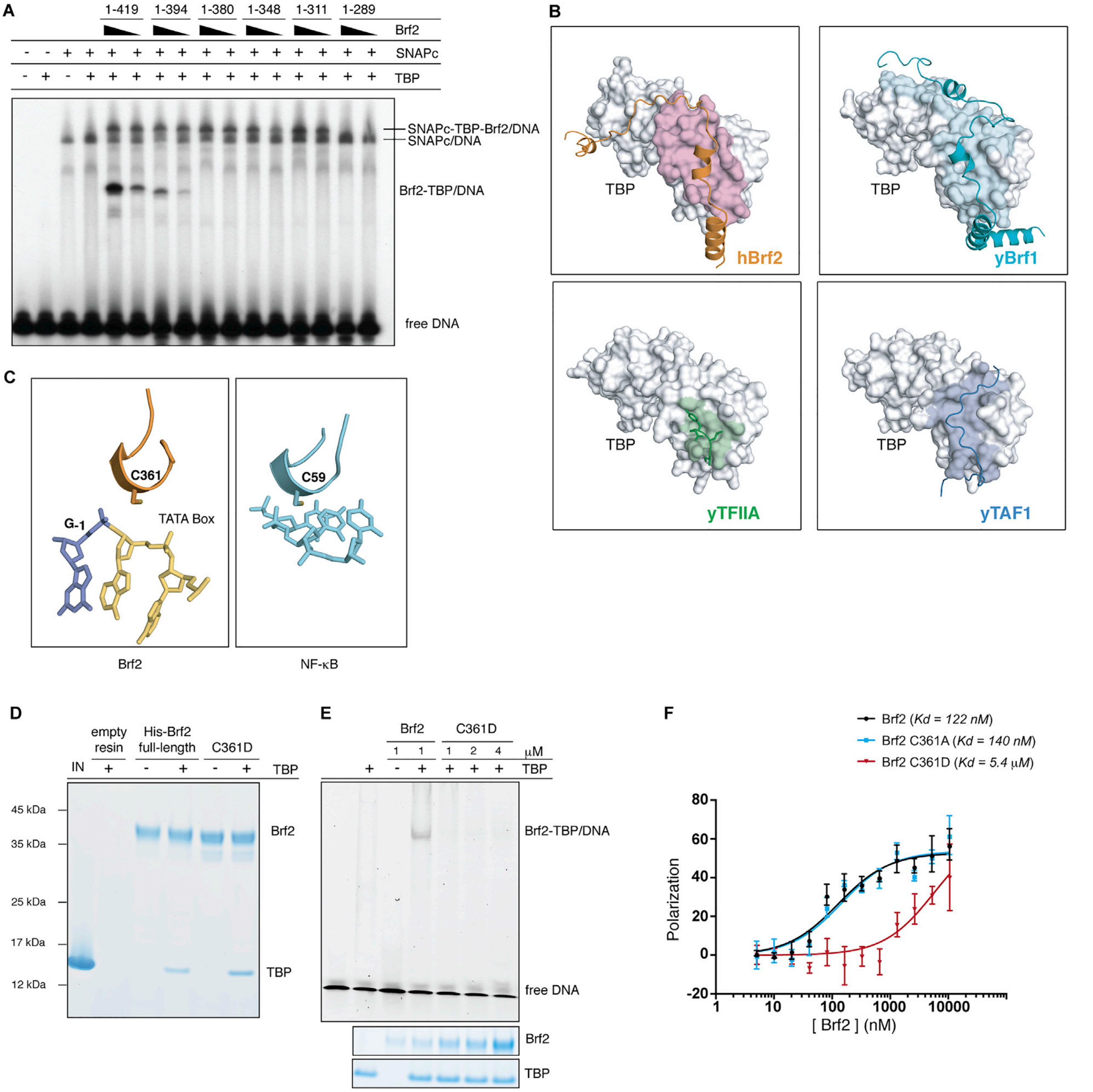

Figure S3. Modular Functions of the Brf2 CTD, Related to Figures 4 and 5

(A) EMSA with serial Brf2 C-terminal deletion mutants showing that the region comprised between residues 289-311 of Brf2 is involved in direct binding to the upstream transcription factor SNAPc.

(B) A conserved surface of TBP is utilized by different TBP associated factors. The TBP surface buried upon interaction with the associated factor is colored in pink for human Brf2 (orange), in cyan for yeast Brf1 (PDB id: 1NGM, turquoise), in green for yeast TFIIA (PDB id: 1NH2, green) and in blue for yeast TAF1 (PDB id: 4B0A, blue).

(C) Structural conservation between Brf2 C361 part of the molecular pin, and C59 part of a short helical motif of the p-50 subunit of the NF-kB transcription factor (PDB id: 1NFK).

(D) Brf2 oxidative-mimic mutation C361D does not hinder Brf2-TBP complex formation in absence of the DNA, as shown by pull-down assay. “IN” indicates the input and “empty resin” the eluted untagged TBP binding non-specifically to the resin.

(E) EMSA shows that formation a functional Brf2-TBP/DNA complex is severely impaired in Brf2 oxidative-mimic mutant C361D. “IN” indicates the input and “empty resin” the eluted untagged TBP binding non-specifically to the resin.

(F) Fluorescence polarization saturation binding assay shows virtually no reduction in affinity of the Brf2 C361A mutant and an approximately 50-fold reduction in affinity of Brf2 C361D mutant for TBP/DNA complexes.

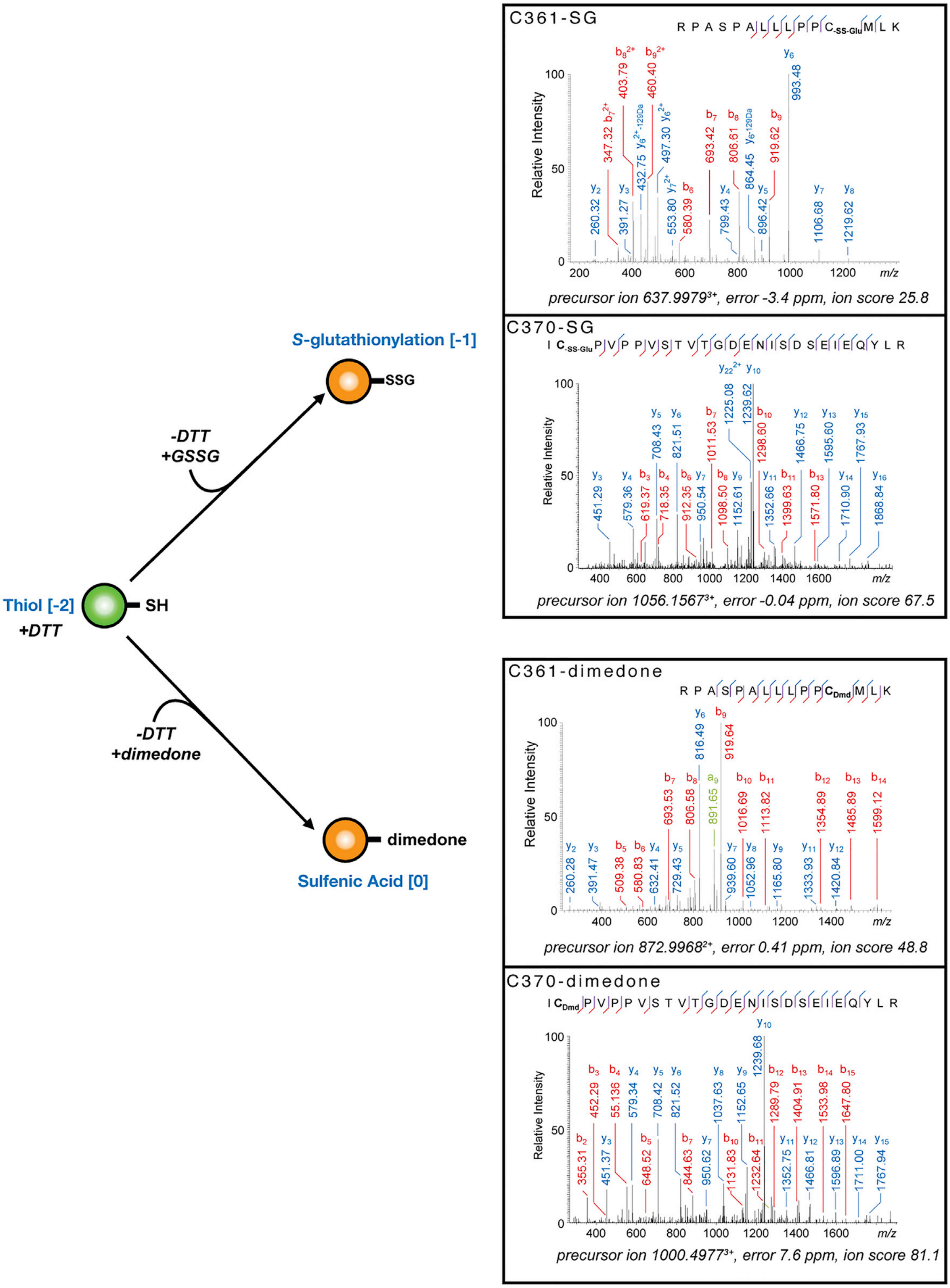

Figure S4. Mass Spectrometry Analysis of Brf2 Redox Modifications, Related to Figure 5

Biologically relevant oxidation states of C361 were confirmed by MS/MS as either unmodified, trapped with glutathione (-SS-Glu) or dimedone (Dmd). The precursor ions, errors and ion scores are indicated below the annotated fragmentation mass spectra. For clarity, only prominent fragment ions are marked.

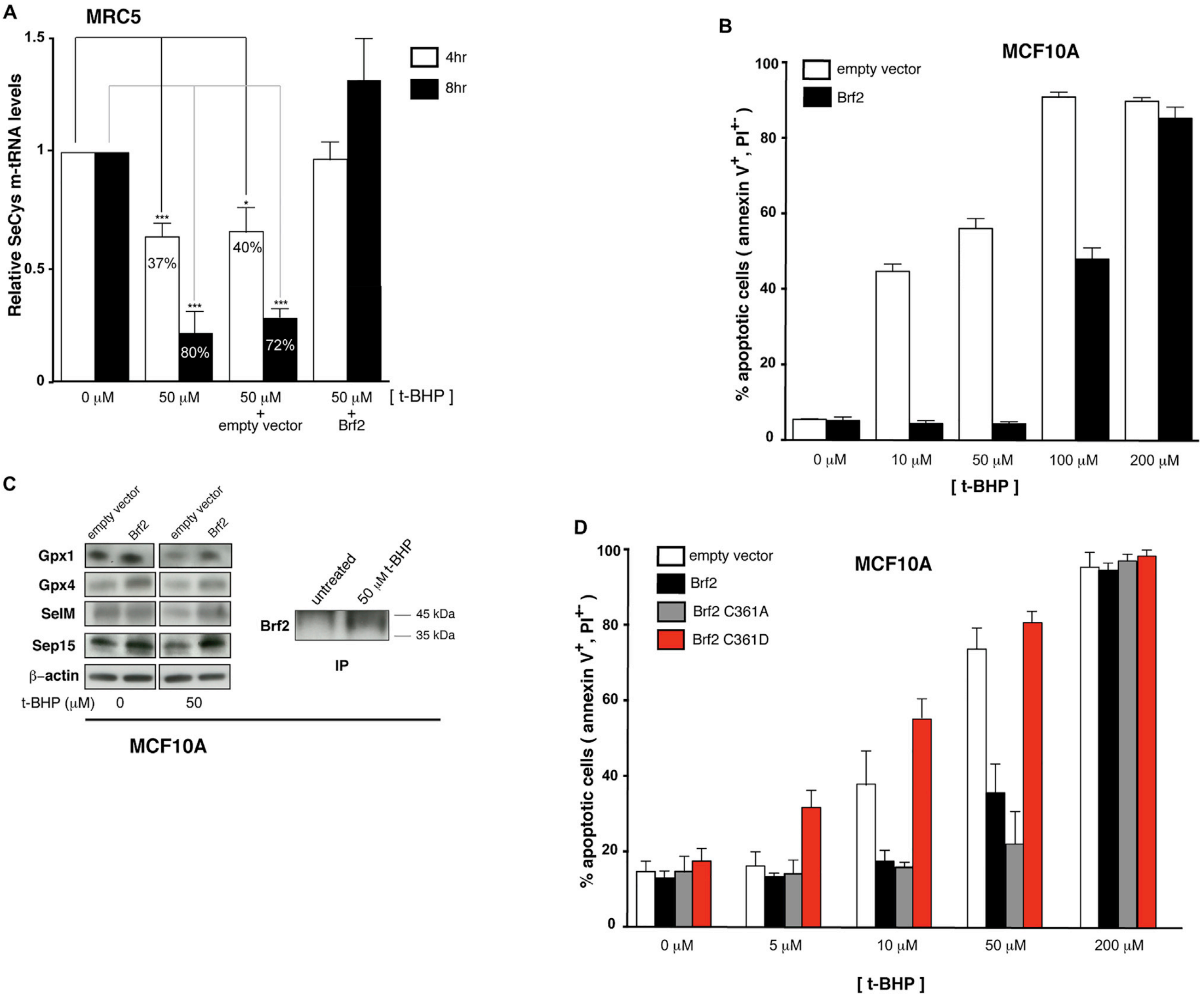

Figure S5. Brf2 is a Redox Sensor in Living Cells, Related to Figures 6 and 7

(A) SeCys m-tRNA levels are reduced during oxidative stress in a Brf2-dependent manner, as monitored via four-leaf clover PCR (Honda et al., 2015). Samples labeled empty vector and Brf2 represent transient over-expressions.

(B) Overexpression of Brf2 in MCF10A cells challenged with t-BHP results in decreased apoptosis as measured by FACs analysis via Annexin V-FITC/PI staining.

(C) Overexpression of Brf2 affects selenoproteins expression levels during oxidative stress in MCF10A cells.

(D) Effects of overexpression of Brf2 and Brf2 mutants on acquired resistance to apoptosis in MCF10A cells as measured by FACs analysis via Annexin V-FITC/PI staining.

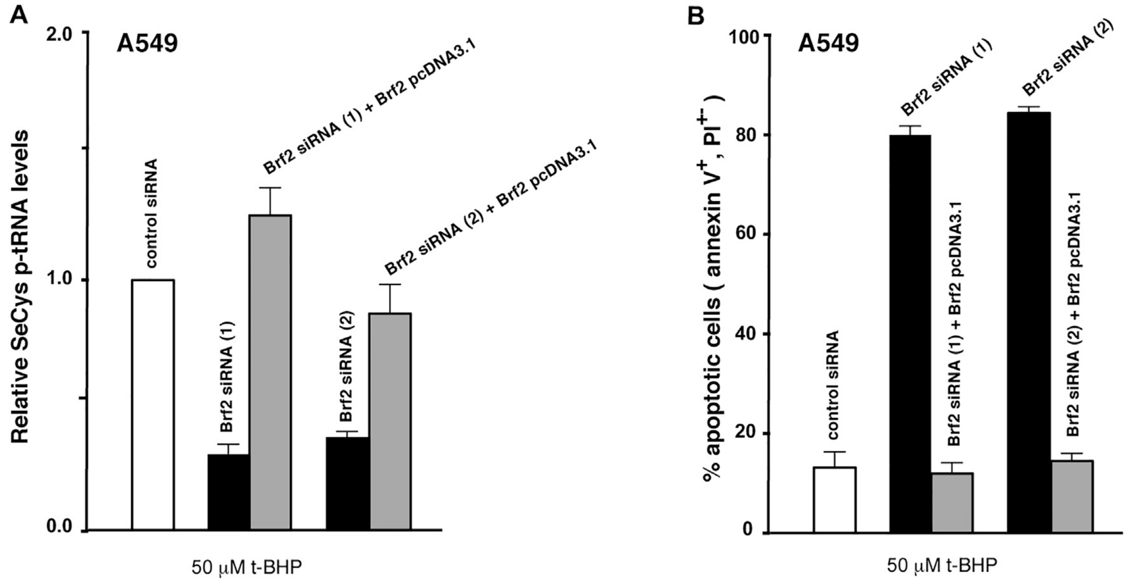

Figure S6. Brf2-Dependent Reduction of SeCys p-tRNA and Enhanced Apoptosis in A549 Cells, Related to Figure 7

(A) Two individual Brf2 siRNAs cause a severe reduction of SeCys p-tRNA in A549 cells challenged with t-BHP, an effect that is fully rescued by concomitant overexpression of a siRNA resistant form of Brf2.

(B) Two individual Brf2 siRNAs elicit a strong sensitization towards t-BHP in A549 cells, an effect that is fully rescued by concomitant overexpression of a siRNA resistant form of Brf2.

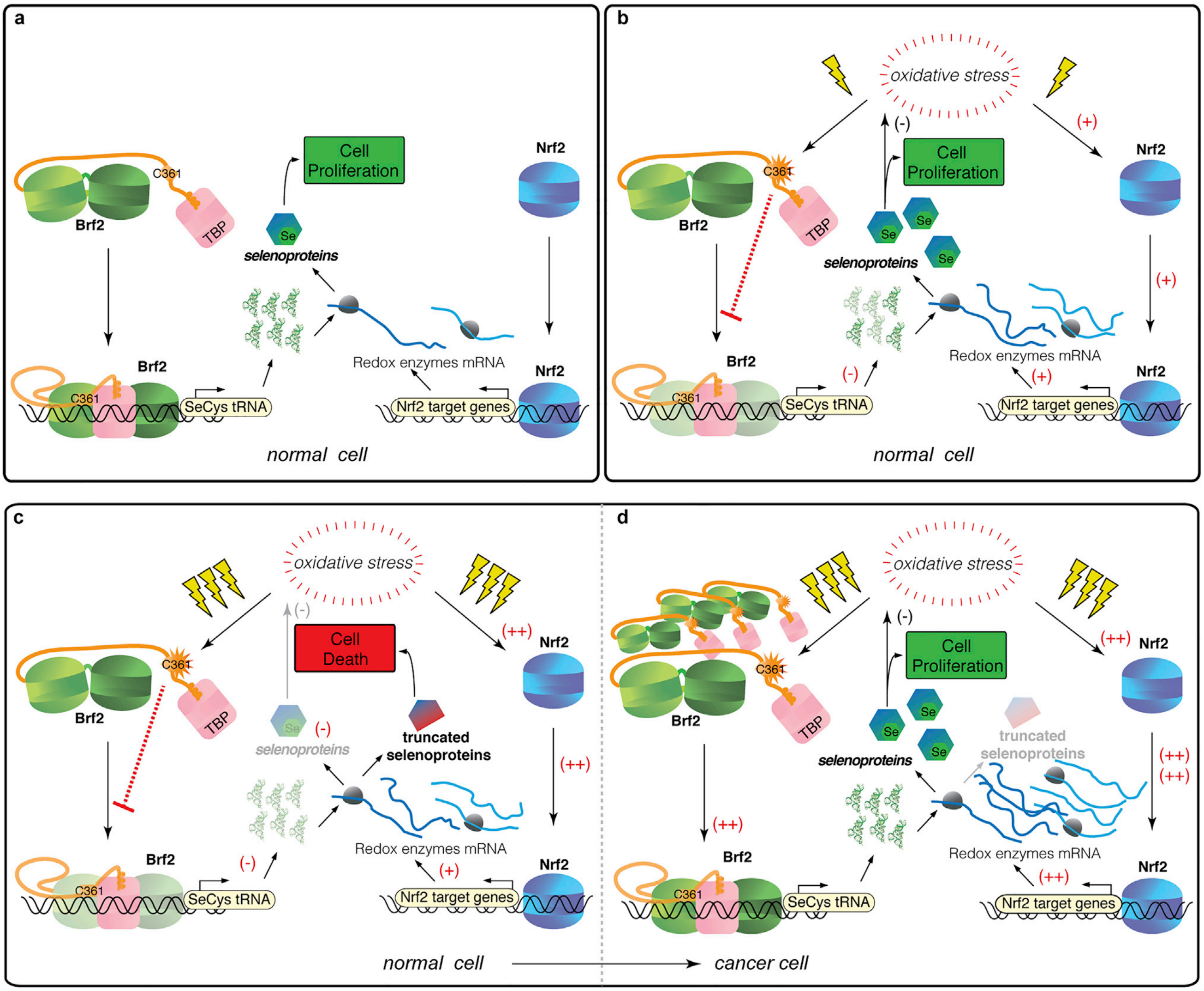

Figure S7. Mechanism of the Redox-Dependent Brf2 Blockade during Oxidative Stress and Carcinogenesis, Related to Figures 1, 2, 3, 4, 5, 6, and 7

During normal growth conditions (A) Brf2- and Nrf2-dependent transcripts are synthesized at basal levels. Upon moderate oxidative stress (B), Nrf2 is activated and Nrf2-dependent transcripts upregulated. Concomitantly, Brf2-dependent transcription, including SeCys tRNAs, is rapidly downregulated via redox-dependent modifications of Brf2. The pre-existing pool of SeCys tRNA is sufficient to sustain synthesis of selenoproteins. Upon prolonged oxidative stress (C), SeCys tRNA levels become limiting while, simultaneously, selenoprotein's mRNAs continue to be highly upregulated by Nrf2. In this scenario, compromised synthesis of selenoproteins drives the cells into apoptosis. In cancer cells (D), the Nrf2 pathway is constitutively activated and contributes to the observed resistance of cancerous cells to higher than normal concentrations of reactive oxygen species. Under these circumstances, Brf2 overexpression is required to overcome the innate redoxdependent blockade, ensuring elevated synthesis of SeCys tRNAs and, ultimately, enabling cancer cells to evade apoptosis under prolonged oxidative stress.

Cell

Supplemental Information

Redox Signaling by the RNA Polymerase III TFIIB-Related Factor Brf2

Jerome Gouge, Karishma Satia, Nicolas Guthertz, Marcella Widya, Andrew James Thompson, Pascal Cousin, Oleksandr Dergai, Nouria Hernandez, and Alessandro Vannini

SUPPLEMENTAL EXPERIMENTAL PROCEDURES

Expression and purification of recombinant proteins.

All proteins were expressed in Rosetta(DE3)pLysS. TB, and TBP-Brf2 complex were both expressed overnight at 20°C with 1 mM IPTG (Isopropyl β-D-1-thiogalactopyranoside) in TB medium. Full-length Brf2 was expressed at 30°C for 4 hours with 1 mM IPTG. Cells were grown at 37°C until the optical density reached 0.6 (A600), then the temperature was reduced and IPTG was then added 30 minutes after. Cells were harvested by centrifugation at 4000g (20 minutes at 4°C) and the pellets stored at -80°C. For crystallization, pellets of Brf2 co-expressed with TBP were re-suspended in 750 mM NaCl, 10 mM imidazole, 50 mM HEPES pH 7.9, 10% glycerol, 5 mM β-mercaptoethanol (buffer A1), supplemented with protease inhibitors tablets (Pierce) and DNase I (20 mg/ml). Cells were lysed through sonication and clarified by centrifugation (14000g for 45 minutes at 4°C). The supernatant was applied onto a 5 mL HisTrap HP column (GE healthcare) and bound proteins were washed with 50 ml of buffer A1 supplemented with 50 mM imidazole, then eluted with buffer B1 (buffer A1 containing 300 mM imidazole). The salt concentration was reduced to 350 mM by diluting the sample with buffer H1 (50 mM HEPES pH 7.9, 10% glycerol, 5 mM DTT). The proteins's samples were loaded onto a 5 mL HiTrap Heparin HP column (GE Healthcare) and eluted with a 375 mM to 1.5 M NaCl gradient in 10 column volumes. At this stage, Brf2-TBP complex was dissociating and the subsequent steps were performed to purify Brf2 in isolation. The fractions containing the protein of interest were pooled and the N-terminal His-tag was cleaved overnight at 4°C with the 3C protease. Imidazole (20 mM) was added to the sample before loading onto a 5 ml HisTrap HP column. The flow-through was concentrated and applied onto a Superdex 200 26/600 column (GE Healthcare). The size exclusion chromatography was performed in a buffer containing 500 mM ammonium acetate, 50 mM HEPES pH 7.9, 10% glycerol and 2 mM DTT (gel filtration buffer). The fractions containing Brf2 were pooled, concentrated, flash-frozen and stored at -80°C. For full-length Brf2, the same protocol was used, with the exception that the tag was not cleaved and the second nickel column step was omitted. For experiments aiming to assess the effect of oxidative modifications on Brf2, DTT was removed from the buffer though a PD-10 desalting column (GE Healthcare) and the protein was immediately flash frozen.

His-TBP pellets were re-suspended in buffer A2 (A1 buffer containing 500 mM NaCl) supplemented with protease inhibitors tablets (Pierce) and DNase I (20 mg/ml). Cell lysis and clarification were performed as mentioned above and the supernatant applied on a 5 ml HisTrap HP column (GE healthcare). The bound proteins were washed with 50 ml of buffer A2 supplemented with 50 mM imidazole, then eluted with buffer B2 (similar to buffer B1 but with 500 mM NaCl). The fractions containing His-TBP were pooled and loaded onto a 5mL HiTrap Heparin HP column (GE Healthcare). The protein was eluted with 500 mM to 2 M NaCl gradient in 10 column volumes. The tag was cleaved overnight at 4°C by addition of the 3C protease. The reaction was then adjusted to 20 mM

imidazole and loaded onto a HisTrap HP column. The flow through was concentrated and loaded onto a Superdex 200 16/600 column (GE Healthcare) in gel filtration buffer. After elution, the protein was concentrated, flash frozen and stored at -80°C. SNAPc was expressed in insect cells and purified as described (Henry et al., 1998).

Oligonucleotides.

All oligonucleotides were purchased from Integrated DNA Technologies. Each single strand was re-suspended in 50 mM Tris pH 8.0, 5 mM $MgCl_2$, 1 mM EDTA and mixed with the complementary strand in an equimolar ratio, heated at 95°C for 3 minutes. then allowed to cool down to room temperature overnight.

Crystallization, data collection and processing.

DNA sequences used for crystallization are described in Figure S1. The complexes were assembled at a final concentration of 60 µM with an equimolar ratio of TBP and Brf2 and 1.2 excess of the double stranded DNA. Crystals were grown by mixing 1 µL of the complexes and 1 µL of the crystallization solution (10-20% PEG 3350, 50-100 mM $MgCl_2$, 2 mM DTT) in hanging drop plates. Seeds were introduced after 3 hours of equilibration. After 5 days, crystals were cryo-protected in a mix of 50% paraffin/50% paratone and flash frozen in liquid nitrogen. Initial data sets were collected at the ESRF (France) on ID29 and ID23eh1. The final high-resolution diffraction data were collected on beamlines I24 (U6) and IO3 (RPPH1 and TRNAU1) at Diamond Light Source (UK). The data were indexed with XDS (Kabsch, 2010), scaled and merged with SCALA (Evans, 2011) from the ccp4 package (Winn et al., 2011). The data were processed using $CC_{1/2}$ and completeness as cut-off criterion (Karplus and Diederichs, 2012).

Structure determination and refinement.

The structure of Brf2 bound to TBP and a U6 promoter was solved by molecular replacement using the TFIIB structure (PDB id: 1C9B) as a search model in PHASER (McCoy et al., 2007). The solution was confirmed by using a 2 ensemble search approach (first TBP and dsDNA then the cyclin repeats of TFIIB). The quality of the resulting electronic density did not allow the manual reconstruction directly after the molecular replacement. To improve the phases, solvent flattening and dummy atom building were carried out with DM (Cowtan, 2010) and ARP/wARP (Langer et al., 2008), respectively. The density modified maps clearly showed α-helices in the cyclin repeats region. Iterative manual building was performed with COOT (Emsley et al., 2010), and BUSTER-TNT (version 2.10.1) was used for refinement (Blanc et al., 2004). The U6-2 structure was used a search model for molecular replacement of RPPH1 and TRNAU1 structures. The quality of the final structures was assessed with MolProbity (Chen et al., 2010). Data collection and refinement statistics are shown in Table S2. The figures were prepared with PyMol (version 1.7.0.5 Schrödinger, LLC). The alignments were

performed with Clustal Omega (Sievers and Higgins, 2014) and prepared with ESpript (Robert and Gouet, 2014).

Electrophoretic mobility shift assay.

The oligonucleotides used in this study are all Cy-5 labelled and based on the U6_2 sequence: 5'-Cy5- ATTTGATTGAAGGGCTTAAAATAGGTGTGACAGTAACC-3' and 5'-GGTTACTGTCACACCTATTTTAAGCCCTTCAATCAAAT-3'. The complexes were assembled in a 25 µL reaction volume in a buffer containing 500 mM ammonium acetate, 50 mM HEPES pH 7.9, 10% glycerol with or without the addition of 5 mM DTT. The complexes were assembled by mixing 1 pmol of the dsDNA with 75 pmol of TBP and 25 pmol of Brf2 and incubated at room temperature for 1 hour. For the EMSAs in Figure 5, 10 pmol of Brf2, from which the DTT had been removed with a PD-10 column, was pre-incubated with either H_2O_2, or oxidised/reduced glutathione (GSSG/GSH) gradients (3 m final) for 20 minutes at room temperature. Then 15 pmol of TBP and 1 pmol of dsDNA (with 1.1 excess of labeled ssDNA) were added to a final volume of 25 µL. The incubation lasted for 20 minutes. For the incubation with iodoacetamide, Brf2 (10 pmol) was first reduced with 0.5 mM DTT for 15 minutes, then the iodoacetamide was added at the final concentration indicated. The reaction was quenched after 30 minutes at room temperature with 50 mM DTT. TBP and the DNA were then added as previously described. For the time course experiment, 10 pmol of Brf2 were incubated at 4°C for different time points, then TBP and the DNA were added. The binding reactions were resolved on a 4% polyacrylamide (37.5:1 acrylamide/bis-acrylamide, 10% glycerol, Tris borate EDTA 1X) gel in 1X Tris- borate EDTA running buffer at 40 mA. The gels were then scanned with a Typhoon FLA 9500 and band quantification was carried out with ImageQuanT TL version 8.1. The EMSA with SNAPc (Figure S3A) was performed as described previously (Saxena et al., 2005).

Pull-downs.

To study the interaction of Brf2 and TBP, 50 µg of Brf2 were loaded onto His SpinTrap columns (GE Healthcare) in presence or in absence of 100 µg of TBP, in a buffer containing 500 mM ammonium acetate, 50 mM HEPES pH 7.9, 10 mM imidazole, 10% glycerol and 2 mM β-mercaptoethanol in a total volume of 400 µL. The incubation was performed at 4°C for 2 hours. The beads were extensively washed with the same buffer supplemented with 50 mM imidazole. The proteins were eluted with 75 µL of the binding buffer containing 300 mM imidazole, boiled and analyzed on a SDS-PAGE.

Fluorescence polarization assay.

The oligonucleotides used for the fluorescence polarization studies, 5'-Alexa488-ATTGAAGGGCTTAAAATAGGTGTGACAG-3' and 5'-GGTTACTGTCACACCTATTTTAAGCCCTTCAATCAAAT-3', were purchased from Integrated DNA Technologies, annealed overnight as previously described. The TBP-DNA complex was assembled

by saturating the oligonucleotides (100 nM final) with 250 mM final of TBP at room temperature for an hour in a volume of 5 µL. A serial dilution of Brf2 (from 10 mM to 5nM) was added and incubated for an extra hour at room temperature in 50 µL total reaction volume. The data were collected at 25°C on a POLARstar Omega plate reader (BMG Labtech), with excitation at 485 nm and emission at 520 nm, and analyzed with MARS Data Analysis Software version 2.10 R3 (BMG Labtech). The binding constants were calculated with GraphPad Prism 6. Three independent experiments were carried out in order to calculate standard deviation.

Mass spectrometry.

Solvents were purchased from Rathburn (Walkerburn, UK). All other reagents were purchased from Sigma unless otherwise stated. All the mass spectrometry experiments were performed with Brf2 full-length carrying a His-Tag at its C-terminus. Prior to treatment, the buffer was exchanged with PD-10 columns (GE Healthcare) to have Brf2 in 50 mM HEPES pH 7.9 and 500 mM ammonium acetate. To check the different oxidative intermediates, Brf2 was incubated at room temperature for 1 hour with a) 5 mM DTT, b) 3 mM of oxidized glutathione or c) overnight with 2 mM dimedone. Solutions of 10-20 µM Brf2 were diluted 3-4 fold with 50 mM triethylammonium bicarbonate/5% acetonitrile and digested directly by addition of 20-50 ng trypsin and incubation for 1 hour at 37°C, followed by a second addition of 20-50 ng trypsin and incubation for a further 3 hours at 37°C. A 5 µL aliquot of each digest was injected directly for LC-MS analysis. Reversed phase chromatography was performed with an HP1200 platform (Agilent). Peptides were resolved on a 75 µm I.D. 15 cm C18 packed emitter column (3 µm particle size; Nikkyo Technos) over 30 minutes using a linear gradient of 96:4 to 50:50 buffer A:B (buffer A: 1% acetonitrile/3% dimethyl sulfoxide/0.1% formic acid; buffer B: 80% acetonitrile/3% dimethyl sulfoxide/0.1% formic acid) at 250 nL/minute. Peptides were ionised by electrospray ionisation with 1.8 kV applied immediately pre-column via a microtee built into the nanospray source. Sample was infused into an LTQ Velos Orbitrap mass spectrometer (Thermo Fisher Scientific) directly from the end of the tapered tip silica column (6-8 µm exit bore). The ion transfer tube was heated to 275°C and the S-lens set to 60%. MS/MS were acquired using data dependent acquisition based on a full 30,000 resolution FT-MS scan with preview mode disabled and internal lock mass calibration against ion 401.922718. The top 10 most intense ions were fragmented by collision-induced dissociation and analyzed with enhanced ion trap scans. Precursor ions with unknown or single charge states were excluded from selection. Automatic gain control was set to 1,000,000 for FT-MS and 30,000 for IT-MS/MS, full FT-MS maximum inject time was 500 ms and normalized collision energy was set to 35% with an activation time of 10 ms. Wideband activation was used to co-fragment precursor ions undergoing neutral loss of up to -20 m/z from the parent ion, including loss of water/ammonia. MS/MS was acquired for selected precursor ions with a single repeat count acquired after 5 seconds delay followed by dynamic exclusion with a 10 ppm mass window for 10 seconds based on a maximal exclusion list of 500 entries. Raw MS/MS data was compiled and interrogated against the swissprot 2011_01 human database, customized to include the

Brf2 construct sequence, with Proteome Discoverer v1.4 and Mascot v2.3. The Mascot search parameters used were: 10 ppm precursor peptide mass error, 0.25 Da fragment mass error, and trypsin specificity with up to two missed cleavages.

Cell culture.

MRC5 and A549 cell lines (ATCC, UK) were maintained and grown in EMEM (ATCC, UK) and Hams F-12 (Kaighn's) media (Life technologies, CA, USA) respectively, and supplemented with 10% (v/v) FBS (Life technologies) and 100 U/ml penicillin and streptomycin (Sigma). MCF10A (ATCC, UK) were maintained in DMEM/F12 (Life technologies, CA, USA) and supplemented with 5% (v/v) donor horse serum (16050122, Life technologies), Insulin (10 µg/ml, I9278, sigma), cholera toxin (100 ng/ml, C8052, Sigma), EGF (20 ng/ml, AF-100-15, Peprotech), Hydrocortisone (0.5 mg/ml, H6909, Sigma) and 100 U/ml penicillin and streptomycin. All cells were grown at 37°C in a 5% CO2 humidified atmosphere. Cells were treated with varying concentrations of t-BHP (Sigma, St Louis, MA, USA) in the conditioned medium and subsequently lysed with RIPA lysis buffer (50 mM Tris pH 8.0, 150 mM NaCl, 1% (v/v) NP40, 0.5% (v/v) Sodium deoxycholate, 2 mM EDTA) containing protease inhibitors (Pierce).

Immunoprecipitation.

Brf2 antibodies (ab17011, Abcam) were coupled to magnetic beads using the DynaBeads Antibody coupling kit (Life Technologies) according to the manufacturer's protocol. After lysis and centrifugation, the protein concentration in the supernatant was measured with Bradford Protein Assay (Bio-Rad) to ensure that the same amount of total proteins was incubated with the beads. The cell lysates were then incubated overnight at 4°C. Immunoprecipitates were extensively washed with lysis buffer and eluted with SDS loading buffer by heating at 50°C for 10 minutes.

Quantitative RT-PCR.

Total RNA was extracted from treated cells with TRIzol reagent (Life technologies) according to manufacturer's instructions. The Quantitect reverse transcription kit (Qiagen, Netherlands) was used to synthesize cDNA following manufacturers instructions, except for a 10 minutes incubation step at 70°C before addition of the polymerase to the reaction. To determine levels of selenocysteine precursors and mature tRNA, RPPH1 (PPH68975A-200, Qiagen), RNA7SK (PPH68975A-200, Qiagen), U6, tRNA Leu and 5S rRNA, syber green assays were performed. Primers 1) 5'-TCAGTGGTCTGGGGTGCAGG-3' and, 2) 5'- GTCCGGTTCGATAAGTAAGATTTAAGGC-3' were used to determine selenocysteine tRNA precursor levels. Primers 1) 5'-GCACCCCAGACCACTGAG GA-3' and Primers 2) 5'-AGCGACAGAGTGGTTCAATTCCAC-3' were used to determine mature selenocysteine tRNA levels. Primers 1) 5'-GGCCATACCACCCTGAACGC-3' and 2) 5'-CAGCACCCGGTATTCCCAGG-3 were used to determine 5S rRNA levels, the reference transcript for all syber green assays (loading control). Primers used to determine levels of U6 were 1) 5'-

GGAACTCGAGTTTGCGTGTCATCCTTGCGC-3 2) 5'-GGAATCTAGAACATATACTAAAATTGGAAC-3' and tRNA Leu were 1) 5'-GTCAGGATGGCCGAGTGGTCTAA-3' 2) 5'-TGTCAGAAGTGGGATTCGAACCCAC-3'. Amplification of cDNA was performed in a 20 µl reaction containing either 1) PerfeCTa SYBR Green FastMix (VWR, Radnor, USA), 0.3 µM of syber green primers mentioned above, and cDNA, (22.5 and 15 ng to detect selenocysteine tRNA precursor and mature levels resepctively and 7.5 ng of cDNA for other RNA levels). RT-PCR was performed with the Applied Biosystems 7900HT Fast Real time PCR system and the samples were subjected to an initial denaturation step at 95°C for 10 minutes, followed by 40-cycles of 95°C for 20s, 60°C for 20s, and 72°C for 20s. All RNA levels were quantified with the relative standard curve method and the Brf1-dependent 5S rRNA as an internal control. Mature selenocysteine tRNA levels were quantified using the comparative Ct method and 5S rRNA as internal control. Prism 6 was used to perform unpaired t-test statistical analysis to determine p values. P values < 0.05 were deemed significant and data shown is from three biological repeats.

Flow cytometry.

Cells were trypsinized and re-suspended in media containing 1 µg mL-1 of Propidium iodide (Sigma), 5 µL per well of FITC-Annexin V (BD Biosciences) and 2.5 mM $CaCl_2$. Cells were then analyzed by flow cytometry on a LSR II SPOC instrument (BD Biosciences).

Western Blot.

Proteins were separated via SDS-PAGE and transferred to a nitrocellulose membrane (GE-Healthcare). The membrane was then blocked for 1 hour in protein-free RotiBlock buffer (Carl Roth, Germany) and then incubated for 1 hour at room temperature with primary antibodies for anti-BRF2 (ab17011, 1:1000, Abcam), anti-beta Actin (ab6276, 1:5000, Abcam) or anti-Glutathione (ab19534, 1:1000, Abcam) diluted in RotiBlock buffer. In the case of anti-GPX1 (ab50427, 1:500, Abcam), anti-GPX2 (ab137431, 1:1000, Abcam), anti-GPX4 (ab125066, 1:1000, Abcam), anti-SEP15 (ab124840, 1:1000, Abcam), anti-SelM (ab133681, 1:1000 Abcam), anti-Nrf2 (ab62352, 1:1000, Abcam) primary incubations were performed overnight at 4°C in 5% (w/v) BSA TBS-T. The membranes were then washed with TBST three times, followed by incubation with either rabbit anti Goat IgG-HRP (sc-2768, 1:5000, Santa Cruz Biotechnology, Santa Cruz USA), ECL peroxidase labelled anti-mouse (NA931, 1:5000, VWR) or anti-rabbit (NA931, 1:5000, VWR), and then developed.

Table S1, Related to Figure 1: Actively transcribed Brf2-dependent genes.

Name of gene	Description	Genome location	Function
RMRP	RNA component of mitochondrial RNA processing endoribonuclease	NC_000009.12 /9p21-p12	Mitochondrial RNA processing
RN7SK	RNA polymerase II regulation	NC_000006.12 /6p12.2	P-TEFb (RNA Pol II elongation) inhibition
RNU6 (U6-1)	Component of the spliceosome, catalytic RNA	NC_000015.10 /15q23	RNA processing
RNU6 (U6-2)	Component of the spliceosome, catalytic RNA	19p13.3	RNA processing
RNU6 (U6-7)	Component of the spliceosome, catalytic RNA	NC_000014.9 /14q12	RNA processing
RNU6 (U6-8)	Component of the spliceosome, catalytic RNA	NC_000014.9 /14q12	RNA processing
RNU6 (U6-9)	Component of the spliceosome, catalytic RNA	NC_000019.10 /19p13.3	RNA processing
RNU6ATAC	Component of the minor spliceosome	NC_000009.12 /9q34.2	RNA processing
RNY1	Component of the ribonucleoproteins Ro, implications in DNA replication	NC_000007.14 /7q36	DNA replication
RNY3	Component of the ribonucleoproteins Ro, implications in DNA replication	NC_000007.14 /7q36	DNA replication
RNY4	Component of the ribonucleoproteins Ro, implications in DNA replication	NC_000007.14 /7q36	DNA replication
RNY5	Component of the ribonucleoproteins Ro, implicated in DNA replication	NC_000007.14 /7q36	DNA replication
RPPH1	Component of the RNase P, implicated in tRNA maturation	NC_000014.9/ 14q11.2	5'-tRNA processing
TRNAU1	Selenocysteine tRNA	NC_000019.10/ 19q13.2-q13.3	Translation of selenoproteins

Table S2, Related to Figure 1: Data collection and refinement statistics

	U6 #2	RPPH1	TRNAU
**Data collection**			
Space group	$P2_12_12_1$	$P2_12_12_1$	$P2_12_12_1$
Cell dimensions			
a, *b*, *c* (Å)	77.3 89.9 102.5	77.7 91.9 102.4	77.0 91.3 102.9
a, *b*, *g* (°)	90.0 90.0 90.0	90.0 90.0 90.0	90.0 90.0 90.0
Resolution (Å)	29.5-1.9 (2.0-1.9) *	30-2.2 (2.3-2.2) *	30-2.7 (2.85-2.7) *
R_{merge}	0.058 (3.19) *	0.069 (1.617) *	0.157 (2.216) *
I/s*I*	11.2 (0.5) *	13.9 (0.8) *	6.9 (0.9) *
Completeness (%)	99.3 (99.3) *	99.7 (98.7) *	98.9 (99.4) *
Redundancy	4.2 (4.2) *	5.3 (4.3) *	4.8 (5.0) *
CC1/2	0.999 (0.287) *	0.999 (0.367) *	0.992 (0.312) *
**Refinement**			
Resolution (Å)	29.5-1.9	29.7-2.2	29.7-2.7
No. reflections	56427	199497	20221
$R_{work/}$ R_{free} (%)	18.6/21.6	23.6/25.8	21.4/24.9
No. atoms			
Protein	3832	3826	3798
DNA	1101	1088	1122
Water	388	201	141
B-factors			
Protein	56.9	68.0	79.8
DNA	79.9	86.3	90.5
Water	58.8	59.4	60.5
R.m.s deviations			
Bond lengths (Å)	0.010	0.009	0.010
Bond angles (º)	0.94	0.96	0.99

*Highest resolution shell is shown in parenthesis.

SUPPLEMENTAL REFERENCES

Bailey, T.L., Boden, M., Buske, F.A., Frith, M., Grant, C.E., Clementi, L., Ren, J., Li, W.W., and Noble, W.S. (2009). MEME SUITE: tools for motif discovery and searching. Nucleic Acids Res *37*, W202-208.

Blanc, E., Roversi, P., Vonrhein, C., Flensburg, C., Lea, S.M., and Bricogne, G. (2004). Refinement of severely incomplete structures with maximum likelihood in BUSTER-TNT. Acta crystallographica Section D, Biological crystallography *60*, 2210-2221.

Chen, V.B., Arendall, W.B., 3rd, Headd, J.J., Keedy, D.A., Immormino, R.M., Kapral, G.J., Murray, L.W., Richardson, J.S., and Richardson, D.C. (2010). MolProbity: all-atom structure validation for macromolecular crystallography. Acta crystallographica Section D, Biological crystallography *66*, 12-21.

Cowtan, K. (2010). Recent developments in classical density modification. Acta crystallographica Section D, Biological crystallography *66*, 470-478.

Emsley, P., Lohkamp, B., Scott, W.G., and Cowtan, K. (2010). Features and development of Coot. Acta crystallographica Section D, Biological crystallography *66*, 486-501.

Evans, P.R. (2011). An introduction to data reduction: space-group determination, scaling and intensity statistics. Acta crystallographica Section D, Biological crystallography *67*, 282-292.

Henry, R.W., Mittal, V., Ma, B., Kobayashi, R., and Hernandez, N. (1998). SNAP19 mediates the assembly of a functional core promoter complex (SNAPc) shared by RNA polymerases II and III. Genes and Development *12*, 2664-2672.

Honda, S., Shigematsu, M., Morichika, K., Telonis, A.G., and Kirino, Y. (2015). Four-leaf clover qRT-PCR: A convenient method for selective quantification of mature tRNA. RNA Biol *12*, 501-508.

Kabsch, W. (2010). Xds. Acta crystallographica Section D, Biological crystallography *66*, 125-132.

Karplus, P.A., and Diederichs, K. (2012). Linking crystallographic model and data quality. Science *336*, 1030-1033.

Langer, G., Cohen, S.X., Lamzin, V.S., and Perrakis, A. (2008). Automated macromolecular model building for X-ray crystallography using ARP/wARP version 7. Nat Protoc *3*, 1171-1179.

McCoy, A.J., Grosse-Kunstleve, R.W., Adams, P.D., Winn, M.D., Storoni, L.C., and Read, R.J. (2007). Phaser crystallographic software. Journal of applied crystallography *40*, 658-674.

Robert, X., and Gouet, P. (2014). Deciphering key features in protein structures with the new ENDscript server. Nucleic Acids Res *42*, W320-324.

Saxena, A., Ma, B., Schramm, L., and Hernandez, N. (2005). Structure-function analysis of the human TFIIB-related factor II protein reveals an essential role for the C-terminal domain in RNA polymerase III transcription. Molecular and cellular biology *25*, 9406-9418.

Sievers, F., and Higgins, D.G. (2014). Clustal Omega, accurate alignment of very large numbers of sequences. Methods Mol Biol *1079*, 105-116.

Winn, M.D., Ballard, C.C., Cowtan, K.D., Dodson, E.J., Emsley, P., Evans, P.R., Keegan, R.M., Krissinel, E.B., Leslie, A.G., McCoy, A.*, et al.* (2011). Overview of the CCP4 suite and current developments. Acta crystallographica Section D, Biological crystallography *67*, 235-242.
